# Exploring the association of ESR1 and ESR2 gene SNPs with polycystic ovary syndrome in human females: a comprehensive association study

**DOI:** 10.1186/s13048-023-01335-7

**Published:** 2024-01-29

**Authors:** Fatima Muccee, Naeem Mahmood Ashraf, Suhail Razak, Tayyaba Afsar, Nadia Hussain, Fohad Mabood Husain, Huma Shafique

**Affiliations:** 1 School of Biochemistry and Biotechnology, University of Punjab, Lahore, 52254 Pakistan; 2 Department of Community Health Sciences, College of Applied Medical Sciences, King Saud University, Riyadh, 11451 Saudi Arabia; 3 Department of Pharmaceutical Sciences, College of Pharmacy, Al Ain University, Al Ain Campus, 64141 Al Ain, United Arab Emirates; 4 AAU Health and Biomedical Research Center, Al Ain University, Abu Dhabi Campus, P. O. Box 112612, Abu Dhabi, United Arab Emirates; 5 Department of Food Science and Nutrition, College of Food and Agriculture Sciences, King Saud University, Riyadh, 11451 Saudi Arabia; 6 Institute of Cellular Medicine, Newcastle University Medical School, Newcastle University Newcastle Upon Tyne, Newcastle upon Tyne, UK

**Keywords:** Polycystic ovary syndrome, Single nucleotide polymorphisms, Coding sequence, Sub-cellular localization, Secondary structure

## Abstract

**Background:**

Polycystic Ovary Syndrome (PCOS) affects a significant proportion of human females worldwide and is characterized by hormonal, metabolic, and reproductive dysfunctions, including infertility, irregular menstrual cycles, acanthosis nigricans, and hirsutism. Mutations in the estrogen receptor genes *ESR1 and ESR2*, involved in normal follicular development and ovulation, can contribute to development of the PCOS. The present study focuses on investigating the potential correlation between single nucleotide polymorphisms (SNPs) of *ESR1* and *ESR2* genes and the incidence of this syndrome.

**Methods:**

For this study, SNPs in *ESR1* and *ESR2* genes were retrieved from the ENSEMBL database and analyzed for their effect on mutated proteins using different bioinformatics tools including SIFT, PolyPhen, CADD, REVEL, MetaLR, I-Mutant, CELLO2GO, ProtParam, SOPMA, SWISS-MODEL and HDDOCK.

**Results:**

All the SNPs documented in the present study were deleterious. All the SNPs except rs1583384537, rs1450198518, and rs78255744 decreased protein stability. Two variants rs1463893698 and rs766843910 in the *ESR2* gene altered the localization of mutated proteins i.e. in addition to the nucleus, proteins were also found in mitochondria and extracellular, respectively. SNPs rs104893956 in *ESR1* and rs140630557, rs140630557, rs1596423459, rs766843910, rs1596405923, rs762454979 and rs1384121511 in *ESR2* gene significantly changed the secondary structure of proteins (2D). SNPs that markedly changed 3D configuration included rs1554259481, rs188957694 and rs755667747 in *ESR1* gene and rs1463893698, rs140630557, rs1596423459, rs766843910, rs1596405923, rs762454979 and rs1384121511 in *ESR2* gene. Variants rs1467954450 (*ESR1*) and rs140630557 (*ESR2)* were identified to reduce the binding tendency of ESRα and β receptors with estradiol as reflected by the docking scores i.e. -164.97 and -173.23, respectively.

**Conclusion:**

Due to the significant impact on the encoded proteins, these variants might be proposed as biomarkers to predict the likelihood of developing PCOS in the future and for diagnostic purposes.

**Supplementary Information:**

The online version contains supplementary material available at 10.1186/s13048-023-01335-7.

## Background

Polycystic Ovary Syndrome (PCOS) is a complicated disorder affecting the female reproductive system. It is characterized by the presence of multiple egg-containing collagen-filled follicles that are arrested during growth, a thick ovarian capsule termed the tunica albuginea, central adiposity, obesity, high luteinizing hormone serum levels, hirsutism, and acne caused by the presence of excess androgens. One of the main concerns for most human females is the infertility associated with PCOS due to anovulatory or oligoovulatory infertility and menstrual irregularities. Even after conception many human females are more prone to miscarriage due to this condition [1–3]. Systemic complications include a higher risk of cardiovascular disease, dyslipidemia and hypertension, insulin resistance and a four-fold increased risk of developing Type 2 diabetes. PCOS is also associated with obstructive sleep apnea, endometrial cancer, depression and lipid abnormalities [4–6].

The risk factors linked with the pathophysiology of PCOS can be categorized into three groups, namely genetic, non-genetic, and hormonal. The PCOS associated genes can be classified into six categories: genes associated with adrenal and ovarian steroidogenesis, including *cytochrome P450, family 11, sub-family A, member 1* (CYP11A1), *cytochrome P450, family 17* (CYP17), *cytochrome P450, family 19* (CYP19) and *cytochrome P450, family 21* (CYP21); genes associated with the effects of steroid hormones such as *androgen receptor gene* (AR) and *sex hormone binding globular protein* (SHBG); genes regulating gonadotropin activity and regulation such as *luteinizing hormone* (LH), *follicle stimulating hormone receptor* (FSHR) and *anti-mullerian hormone* (AMH); genes linked to the activity and release of insulin such as *calpain 10* (CAPN10), *insulin receptor substrate-1* (IRS-1), *insulin receptor substrate-2* (IRS-2) and *insulin* (INS) etc. [3, 7–10]. Additionally Environmental factors that contribute to PCOS such as environmental endocrine disruptors (EEDs), obesity, and diet, have not been extensively documented [11]. In addition, hormonal factors such as the presence of hyperandrogenism and insulin resistance are also considered to contribute towards PCOS [12, 13].

The ESR1 and ESR2 genes comprise of 8 and 9 exons, respectively. Their chromosomal locations are 6q25.1 and 14q23.1, respectively [14]. *ESR1 and ESR2* genes encode for ESRα and ESRβ proteins which function as binding sites for estradiol during the process of follicle development and ovulation. Estradiol is a determinant of follicle quality [15]. ESRα expression occurs in interstitial, thecal and granulosa cells (GC) of developing antral follicles while ESRβ is only expressed in GC part of follicle. Both these receptor proteins have spliced isoform i.e. three and four for ESRα (ESRα66, ESRα46 and ESRα36) and ESRβ (ESRβ1 to ESRβ5), respectively [16, 17]. Under normal conditions, estradiol binds with ESRα and β receptors and acts synergistically with follicle stimulating hormone (FSH) to upregulate the expression of steroidgenic hormones including LHR receptor resulting in prominent preovulatory follicle selection and ovulation [18]. Hence, estradiol plays vital role in normal follicle development which is strongly related with ESRα and ESRβ proteins expression [19]. These two receptors play a vital role in regulating estrogen functions and dimerize to control the transcription of several downstream genes involved in physiological ovarian functions [17, 20, 21]. ESRs expression has been found to be increased in epithelium and stroma during proliferative stage of female reproductive cycle [22, 23].

**Fig. 1:**
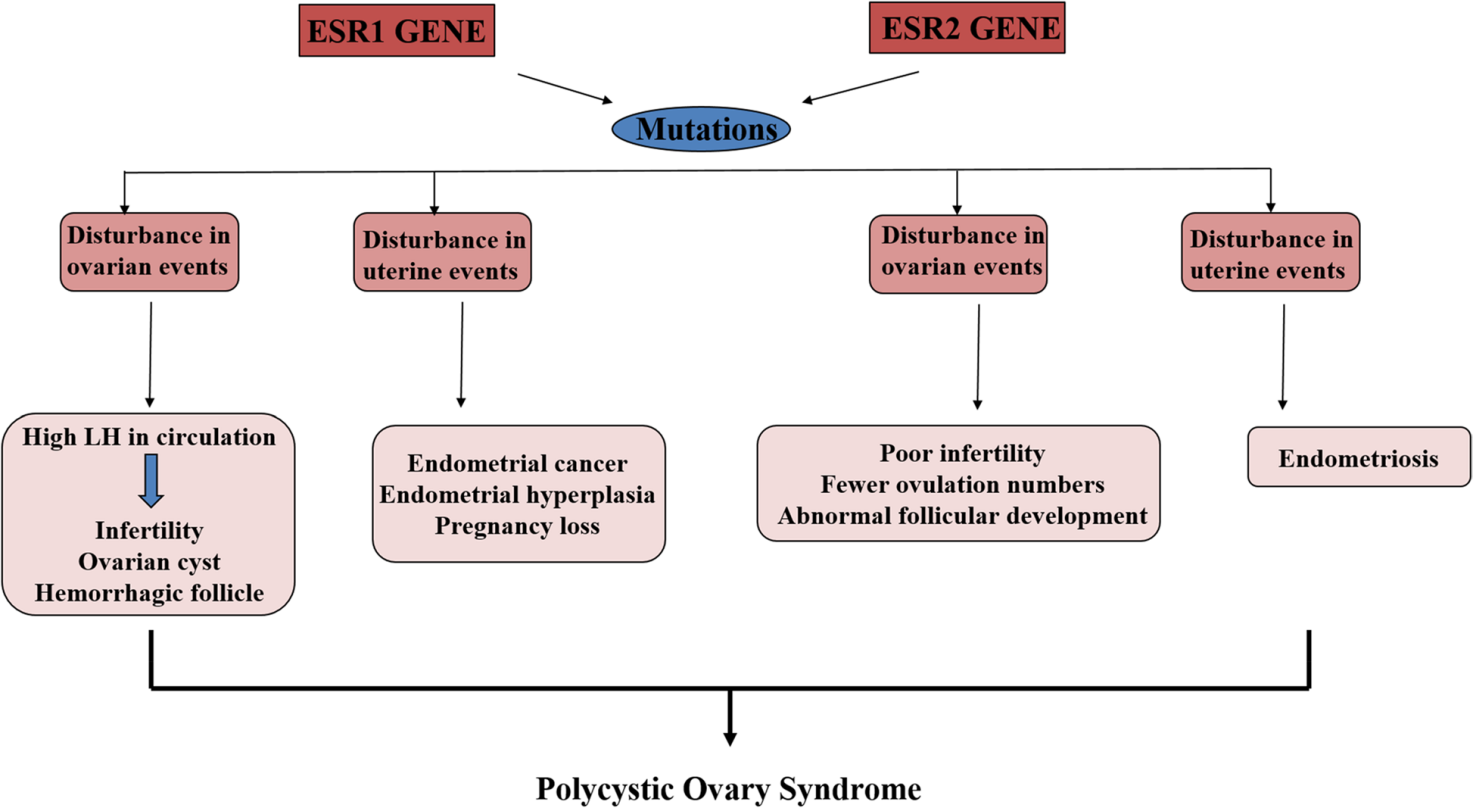
Connection of ESR1 and ESR2 genes mutations and PCOS in human females [17]

Literature also supports the link between ESRs polymorphisms and PCOS like single nucleotide variants of ESRα rs9340799 and rs1999805 have been found to be associated with PCOS patients of Pakistan and China, respectively [24, 25]. An ESRα associated polymorphism rs2234693 has been reported to be found more frequently in PCOS patients than in normal controls [26]. Connection between ESR1 and 2 genes mutations and PCOS is explained in Fig. 1.

Keeping in view the above mentioned association of ESRα and β receptors with PCOS clinical manifestations, these receptor proteins might be considered as most significant markers of PCOS. Any mutations in the ESRα and β encoding genes may disrupt the normal development of follicles, resulting in polycystic ovaries. The present study aimed to identify SNPs in *ESR1 and 2* genes that may lead to abnormal development of ESR α and β receptors. These SNPs could potentially serve as biomarkers for the prognosis and diagnosis of PCOS.

## Methodology

### Retrieving coding sequences of genes and reported SNPs from ENSEMBL

To retrieve the coding sequences of *ESR1* (ENST00000206249.8) and *ESR2* (ENST00000341099.6) genes and the SNPs, ENSEMBL database (https://asia.ensembl.org/index.html, accessed on 1 September 2022) was explored. SNPs were incorporated in normal CDS of genes to generate mutated sequences. The coding sequences of normal genes are shown (Supplementary data Table 1). Stop gained, missense and frame shift mutations retrieved and addressed in present study are described in detail (Table 1).

**Table 1:** Single nucleotide polymorphisms (SNPs) in ESR1 and ESR2 genes associated with PCOS addressed in present study

#	SNPs ID	Consequence type	Position of codon	Nucleotide change	Amino acid change	SIFT	PolyPhen	CADD	REVEL	MetaLR
**ESR1**										
1	rs1583384537	Missense	22	GAG > AAG	E > K	0	0.994	32	0.596	0.88
2	rs1554259481	Stop gained	61	GAG > TAG	E > *	-	-	-	-	-
3	rs104893956	Stop gained	157	CGA > TGA	R > *	-	-	-	-	-
4	rs761613029	Missense	218	GAC > AAC	D > N	0.04	1	31	0.648	0.889
5	rs778449608	Missense	247	GAA > AAA	E > K	0	0.995	32	0.847	0.883
6	rs866869178	Missense	259	CGA > CAA	R > Q	0	0.97	31	0.826	0.946
7	rs188957694	Missense	269	CGC > CCC	R > P	0.01	0.947	32	0.803	0.904
8	rs755667747	Frame shift	293	CCA > CA	P > X	-	-	-	-	-
9	rs1467954450	Frame shift	325	CCG > CC	P > X	-	-	-	-	-
10	rs1584799119	Missense	374	GAT > AAT	D > N	0.01	0.992	31	0.918	0.569
11	rs1131692059	Missense	394	CGC > CAC	R > H	0	0.999	31	0.971	0.969
12	rs762742833	Stop gained	477	CGA > TGA	R > *	-	-	-	-	-
13	rs758798083	Missense	519	AAC > GAC	N > D	0.01	0.955	32	0.909	0.923
14	rs1253340312	Frame shift	570	ACT > AC	T > X	-	-	-	-	-
15	rs1436999383	Stop gained	587	GAG > TAG	E > *	-	-	-	-	-
**ESR2**										
1	rs1463893698	Stop gained	72	CAG > TAG	Q > *	-	-	-	-	-
2	rs140630557	Stop gained	133	TGC > TGA	C > *	-	-	-	-	-
3	rs1450198518	Missense	171	GCC > TCC	A > S	0	1	31	0.874	0.943
4	rs754945292	Missense	187	GCT > GGT	A > G	0	0.967	33	0.845	0.909
5	rs1596423459	Stop gained	201	TGC > TGA	C > *	-	-	-	-	-
6	rs766843910	Stop gained	237	GAG > TAG	E > *	-	-	-	-	-
7	rs1596405923	Frame shift	316	ATT > AT	I > X	-	-	-	-	-
8	rs762454979	Frame shift	331	TTG > TG	L > X	-	-	-	-	-
9	rs1384121511	Stop gained	347	TCA > TAA	S > *	-	-	-	-	-
10	rs1249242790	Missense	380	CTC > CCC	L > P	0	0.998	31	0.971	0.959
11	rs1414263985	Missense	396	GAA > AAA	E > K	0	1	32	0.952	0.988
12	rs78255744	Missense	408	TCC > TTC	S > F	0	0.999	33	0.96	0.956
13	rs768924970	Missense	454	CGC > TGC	R > C	0	1	32	0.962	0.978
14	rs1257844897	Frame shift	466	AGG > AG	R > X	-	-	-	-	-
15	rs200502775	Stop gained	525	CAG > TAG	Q > *	-	-	-	-	-

### Translation of normal and mutated genes sequences

ExPaSy tool (https://web.expasy.org/translate/, accessed on 1 September 2022) was employed to convert nucleotide sequences of *ESR1 and ESR2* genes into amino acid sequences [27].

### Assessment of missense SNPs deleteriousness

To assess the deletriousness of non-synonymous missense mutations, five tools were employed. i. e. Sorting Intolerant from Tolerant (SIFT) (https://sift.bii.a-star.edu.sg, accessed on 1 September 2022), Polymorphism Phenotyping (PolyPhen) (https://genetics.bwh.harvard.edu/pph2/, accessed on 1 September 2022), Combined Annotation Dependent Depletion (CADD) (https://cadd.gs.washington.edu/snv, accessed on 1 September 2022), Rare Exome Variant Ensemble Learner (REVEL) and Meta Logistic Regression (MetaLR) [28–32].

### Effect of missense SNPs on stability of mutated proteins

To determine the effect of missense SNPs on stability of mutated proteins, I-Mutant suit (https://folding.biofold.org/i-mutant/i-mutant2.0.html, accessed on 7 September 2022) was used. Gibbs free energy (DGG) and reliability index (RI) were measured. Value of DGG above zero shows increase while below zero represents decrease in stability of mutated proteins [33].

### Physicochemical properties prediction

Physicochemical properties like number of amino acids, molecular weight, isoelectric point (pI), half-life, extinction coefficient, aliphatic index, instability index and grand average of hydropathicity (GRAVY) were determined for the normal as well as mutated proteins using ProtParam tool (https://web.expasy.org/protparam/, accessed on 7 September 2022).

### Sub-cellular localization prediction

To assess the SNPs effect on sub-cellular localization of mutated proteins, CELLO2GO (https://cello.life.nctu.edu.tw/cello2go/, accessed on 7 September 2022) tool was used [34].

### Secondary structure prediction

To predict the effect of SNPs on 2D of mutated protein, SOPMA secondary structure predicted method (https://npsa-prabi.ibcp.fr/cgi-bin/npsa_automat.pl?page=/NPSA/npsa_sopma.html, accessed on 7 September 2022) was employed. Using this method, percentage of alpha helix, extended strand, beta turn and random coil was determined for all the SNPs containing amino acid sequences and results were compared with the normal protein [35]. Disordered residues were assessed in normal and mutated sequences using protein disorder prediction server i.e. PrDOS (https://prdos.hgc.jp/cgi-bin/result.cgi?ppid=376231p1d1662706327, accessed on 13 September 2022). This tool helped to predict the effect of SNPs on number of disordered regions and number of residues exhibiting disorder. The results were obtained in the form of disorder profile plot and sequence of proteins with disordered regions highlighted in red [36].

### Three dimensional structure prediction and validation

To detect the effect of SNPs on 3D configuration of proteins, homology modelling server SWISS-MODEL (https://swissmodel.expasy.org, accessed on August 2023) was used. The pdb structure derived using this tool was validated through ERRAT and PROCHECK (https://saves.mbi.ucla.edu/results?job=1072813&p=errat, accessed on 26 August 2023) tools [37]. In ERRAT validation, the quality was calculated for pdb structures of normal and mutant proteins [38]. On the other hand, in PROCHECK validation, G-value and Ramachandran plots were predicted for the normal and mutant cases. Ramachandran plots helped us in assessment of amino acids in most favored, additional allowed, generously allowed and disallowed regions [39].

### Docking analysis

To analyze the effect of SNPs on interaction of mutated forms of ESR1 and ESR2 with native form of estradiol hormone, docking was performed using web server for protein–protein docking i.e. HDOCK server (hdock.phys.hust.edu.cn, accessed on August 2023). The estradiol pdb structure was uploaded as input receptor molecule while the mutated structures of ESR1 and ESR2 were uploaded as ligands. Docking score, confidence score and ligand RMSD values were recorded.

## Results

### Deleteriousness of SNPs

The present study has documented a total of fifteen SNPs (four stop-gained and eleven missense) of the ESR1 gene and fifteen SNPs (six stop-gained and nine missense) of the ESR2 gene. Deleteriousness analysis of the missense SNPs showed the pathogenicity of all the missense mutations addressed in present study (Table 1).

### Analysis of SNPs effect on stability of mutated proteins

To determine the effect of SNPs on stability of mutated proteins, I-Mutant tool was used. It was found that in case of *ESR1* gene, two SNPs i.e. rs1583384537 and rs1584799119 increased stability of mutated proteins as compared to remaining polymorphisms which had decreasing effect. On the other hand, in case of *ESR2* gene, two out of six missense mutations i.e. rs1450198518 and rs78255744 showed increasing effect while remaining four SNPs reduced the mutated proteins stability (Table 2).

**Table 2:** Effect of SNPs on stability of mutated proteins predicted using I-Mutant at 25°C and pH = 7

#	SNP ID	RI	DDG (kcal/mol)	Stability
ESR1				
1	rs1583384537	0	0.03	increase
2	rs761613029	6	-0.10	decrease
3	rs778449608	8	-1.01	decrease
4	rs866869178	7	-0.77	decrease
5	rs188957694	4	-1.28	decrease
6	rs1584799119	2	0.17	increase
7	rs1131692059	8	-1.10	decrease
8	rs758798083	2	-0.21	decrease
ESR2				
1	rs1450198518	6	0.28	increase
2	rs754945292	6	-0.83	decrease
3	rs1249242790	7	-1.71	decrease
4	rs1414263985	5	-0.10	decrease
5	rs78255744	3	0.23	increase
6	rs768924970	2	-0.23	decrease

*RI* Reliability index, *DDG* Gibbs free energy value, calculated by formula DG (new protein) – DG (wild type)

### Analysis of SNPs effect on physicochemical properties of mutated proteins

In case of ESR1 gene, six out of fifteen SNPs i.e. rs1554259481, rs104893956, rs755667747, rs1467954450, rs762742833 and rs1253340312 altered physicochemical properties of mutated proteins considerably. In these six SNPs, deviation in number of amino acids from normal value of 649 was observed to be 90, 164, 318, 369, 520 and 535, respectively. Lowest value of pI was observed to be 4.73 in case of rs104893956 while the highest (6.67) was found to be caused by SNP rs755667747. SNPs rs1554259481, rs104893956, rs755667747, rs1467954450, rs762742833 and rs1253340312 changed the extinction coefficient from normal value of 62,520 M^−1^cm^−1^ to 8940, 14,900, 35,340, 36,830, 56,560 and 50,725 M^−1^cm^−1^, respectively. Only two SNPs rs755667747 and rs1467954450 changed the aliphatic index from normal value of 73.70 to 51.04 and 57.99, respectively. Only slight variation was observed in aliphatic index in cases of all other SNPs. The only SNP rs1554259481 reduced the instability index of mutated protein to 17.52 from normal 42.52. Half-life remained unaffected in all the cases. Highest alteration in GRAVY was observed in case of SNPs rs755667747 (-0.820) and rs1467954450 (-0.727) as compared to normal value of -0.499 (Table 3).

**Table 3:** Prediction of effect of SNPs on physicochemical properties of mutated proteins

#	SNP ID	No. of amino acids	Mol. Wt	pI	Half life (hours)	Ext. coefficient (M^−1^cm^−1^)	Aliphatic index	Instability index	GRAVY
ESR1									
1	Normal	649	72,431.96	5.48	30	62,520	73.70	42.52	-0.499
2	rs1583384537	649	72,431.01	5.56	30	62,520	73.70	42.75	-0.500
3	rs1554259481	90	9722.94	5.20	30	8940	73.89	17.52	-0.461
4	rs104893956	164	17,655.67	4.73	30	14,900	67.93	41.73	-0.496
5	rs761613029	649	72,430.97	5.52	30	62,520	73.70	42.52	-0.499
6	rs778449608	649	72,431.01	5.56	30	62,520	73.70	42.50	-0.500
7	rs866869178	649	72,403.90	5.43	30	62,520	73.70	41.75	-0.497
8	rs188957694	649	72,372.88	5.43	30	62,520	73.70	41.90	-0.494
9	rs755667747	318	35,092.17	6.67	30	35,340	51.04	46.66	-0.820
10	rs1467954450	369	40,674.70	6.22	30	36,830	57.99	46.27	-0.727
11	rs1584799119	649	72,430.97	5.52	30	62,520	73.70	42.77	-0.499
12	rs1131692059	649	72,412.91	5.47	30	62,520	73.70	42.20	-0.497
13	rs762742833	520	58,068.64	5.31	30	56,560	69.27	43.04	-0.537
14	rs758798083	649	72,432.94	5.43	30	62,520	73.70	42.02	-0.499
15	rs1253340312	535	60,744.21	6.16	30	50,725	75.50	45.06	-0.554
16	rs1436999383	640	71,529.98	5.56	30	62,520	73.97	42.49	-0.507
ESR2									
1	Normal	530	59,216.33	8.81	30	63,590	83.34	55.32	-0.288
2	rs1463893698	71	7738.54	5.28	30	8940	64.51	68.91	-0.432
3	rs140630557	132	14,699.48	7.86	30	21,555	70.83	66.53	-0.595
4	rs1450198518	530	59,232.33	8.81	30	63,590	83.15	55.32	-0.293
5	rs754945292	530	59,218.30	8.81	30	63,590	82.96	54.79	-0.297
6	rs1596423459	200	22,176.86	8.71	30	33,390	59.95	63.83	-0.595
7	rs766843910	236	26,452.90	9.21	30	36,745	58.64	65.47	-0.644
8	rs1596405923	338	37,655.09	9.24	30	58,995	68.40	61.82	-0.462
9	rs762454979	338	37,877.39	9.20	30	53,370	71.83	59.52	-0.445
10	rs1384121511	346	38,859.68	9.02	30	53,370	73.27	60.37	-0.394
11	rs1249242790	530	59,216.28	8.81	30	63,590	82.42	56.15	-0.303
12	rs1414263985	530	59,231.39	8.91	30	63,590	83.15	55.32	-0.293
13	rs78255744	530	59,278.40	8.81	30	63,590	82.96	54.15	-0.290
14	rs768924970	530	59,148.27	8.84	30	63,715	82.23	56.13	-0.295
15	rs1257844897	478	53,614.00	8.94	30	67,475	84.46	53.59	-0.231
16	rs200502775	524	58,533.64	8.81	30	63,590	84.29	53.95	-0.260

In case of ESR2 gene, eight out of fifteen SNPs i.e. rs1463893698, rs140630557, rs1596423459, rs766843910, rs1596405923, rs762454979, rs1384121511 and rs1257844897 altered the number of amino acids i.e. 71, 132, 200, 236, 338, 346 and 478, respectively as compared to normal 530 amino acids. Not a single SNP effected the half-life of mutated proteins. Lowest and highest deviations in normal value of pI i.e. 8.81 were observed in cases of SNPs rs1463893698 (5.28) and rs1596405923, rs766843910, rs762454979 and rs1384121511 (9.24, 9.21, 9.20 and 9.02, respectively). Extinction coefficients of mutated proteins were observed to be altered greatly in cases of mutations rs1463893698 (8940 M^−1^cm^−1^), rs140630557 (21,555 M^−1^cm^−1^), rs1596423459 (33,390 M^−1^cm^−1^) and rs766843910 (36,745 M^−1^cm^−1^). Six SNPs i. e. rs1463893698 (64.51), rs140630557 (70.83), rs1596423459 (59.95), rs766843910 (58.64), rs1596405923 (68.40) and rs762454979 (71.83) caused considerable change in aliphatic index of mutated proteins. Variation in instability index was observed in cases of SNPs rs1463893698 (68.91), rs140630557 (66.53), rs1596423459 (63.83), rs766843910 (65.47), rs1596405923 (61.82) and rs1384121511 (60.37). Mutations rs766843910 and rs1257844897 showed highest and lowest deviations i.e. -0.644 and -0.231, respectively in normal value (-0.288) of GRAVY.

### Analysis of SNPs effect on sub-cellular localization of mutated proteins

The sub-cellular localization prediction revealed that in case of *ESR1* gene, only one SNP rs1554259481 altered the localization of mutated protein from nuclear to nuclear and cytoplasmic (Supplementary data Fig. S1).

In case of ESR2 gene, the localization remained unaffected. While in case of ESR2 gene, two SNPs i.e. rs1463893698 and rs766843910 changed the localization of mutated proteins from normal location (nuclear) to nuclear: mitochondrial and nuclear: extracellular, respectively (Table 4, Supplementary data Fig. S2).

**Table 4:** Effect of SNPs on stability of mutated protein predicted using I-Mutant

Case #	Docking score	Confidence score	Ligand RMSD (Å)
ESR1			
Normal	-321.57	0.9687	59.59
Case 1	-312.34	0.9626	51.40
Case 2	-250.57	0.8820	68.86
Case 3	-227.19	0.8240	48.47
Case 4	-304.62	0.9566	52.44
Case 5	-292.53	0.9453	54.47
Case 6	-263.81	0.9069	62.42
Case 7	-277.91	0.9281	59.18
Case 8	-242.20	0.8634	56.26
Case 9	-164.97	0.5743	68.52
Case 10	-260.94	0.9019	58.70
Case 11	-228.87	0.8288	277.52
Case 12	-260.94	0.9019	58.70
Case 13	-244.96	0.8698	69.04
Case 14	-285.93	0.9381	57.60
Case 15	-307.44	0.9589	59.40
ESR2			
Normal	-275.18	0.9244	74.26
Case 1	-186.57	0.6751	56.00
Case 2	-173.23	0.6141	47.43
Case 3	-275.18	0.9244	74.28
Case 4	-273.60	0.9222	53.76
Case 5	-252.81	0.8866	44.89
Case 6	-260.64	0.9014	55.11
Case 7	-264.65	0.9083	24.63
Case 8	-259.86	0.9000	79.69
Case 9	-267.52	0.9130	24.85
Case 10	-260.94	0.9019	58.70
Case 11	-276.33	0.9260	53.74
Case 12	-270.34	0.9173	74.17
Case 13	-275.73	0.9252	53.97
Case 14	-292.51	0.9453	44.92
Case 15	-275.18	0.9244	74.26

### Analysis of SNPs effect on secondary structures of mutated proteins

Effect of SNPs on different aspects of 2D structure of mutated proteins i.e. alpha helix, extended strand, beta turn, random coil, number of disordered regions and number of disordered amino acids have been observed. It was found that in case of *ESR1* gene, all the SNPs altered 2D structural properties of proteins. The highest and lowest deviations in normal values were observed to be 41.76% (rs1583384537 and rs1584799119) and 25.00% (rs104893956), respectively in case of alpha helix, 7.85% (rs1253340312) and 14.02% (rs104893956), respectively in case of extended strand, 3.08% (rs762742833) and 10.37% (rs104893956), respectively in case of beta turn and 41.60% (rs188957694) and 52.70% (rs1131692059), respectively in case of random coil. All the SNPs except rs1583384537 were found to alter the number of disordered regions and the number of amino acids in disordered regions (Supplementary data Table 2). The highest and lowest deviations from the normal values of number of disordered regions (137) and number of amino acids in disordered regions (7) were observed to be 289 and 11 in case of SNP rs1131692059 and 23 and 2 in case of rs1554259481 mutation, respectively (Supplementary data Figs. S3 and S4).

In case of *ESR2* gene, all the mutations were observed to change the secondary structure of mutated proteins. The highest and lowest alterations in normal values were observed to be 25.15% (rs762454979) and 0% (rs1463893698), respectively in case of alpha helix, 16.19% (rs1463893698) and 7.17% (rs78255744), respectively in case of extended strand, 4.24% (rs766843910) and 1.52% (rs140630557), respectively in case of beta turn and 80.28% (rs1463893698) and 51.05% (rs1257844897), respectively in case of random coil.

The highest and lowest deviations from the normal values of number of disordered regions (177) and number of amino acids in disordered region (7) were observed to be 189 and 7 in case of SNP rs1414263985 and 28 and 2 in case of SNP rs1463893698, respectively (Supplementary data Figs. S5 and S6).

### Analysis of SNPs effect on 3D structure of mutated proteins

**Fig. 2:**
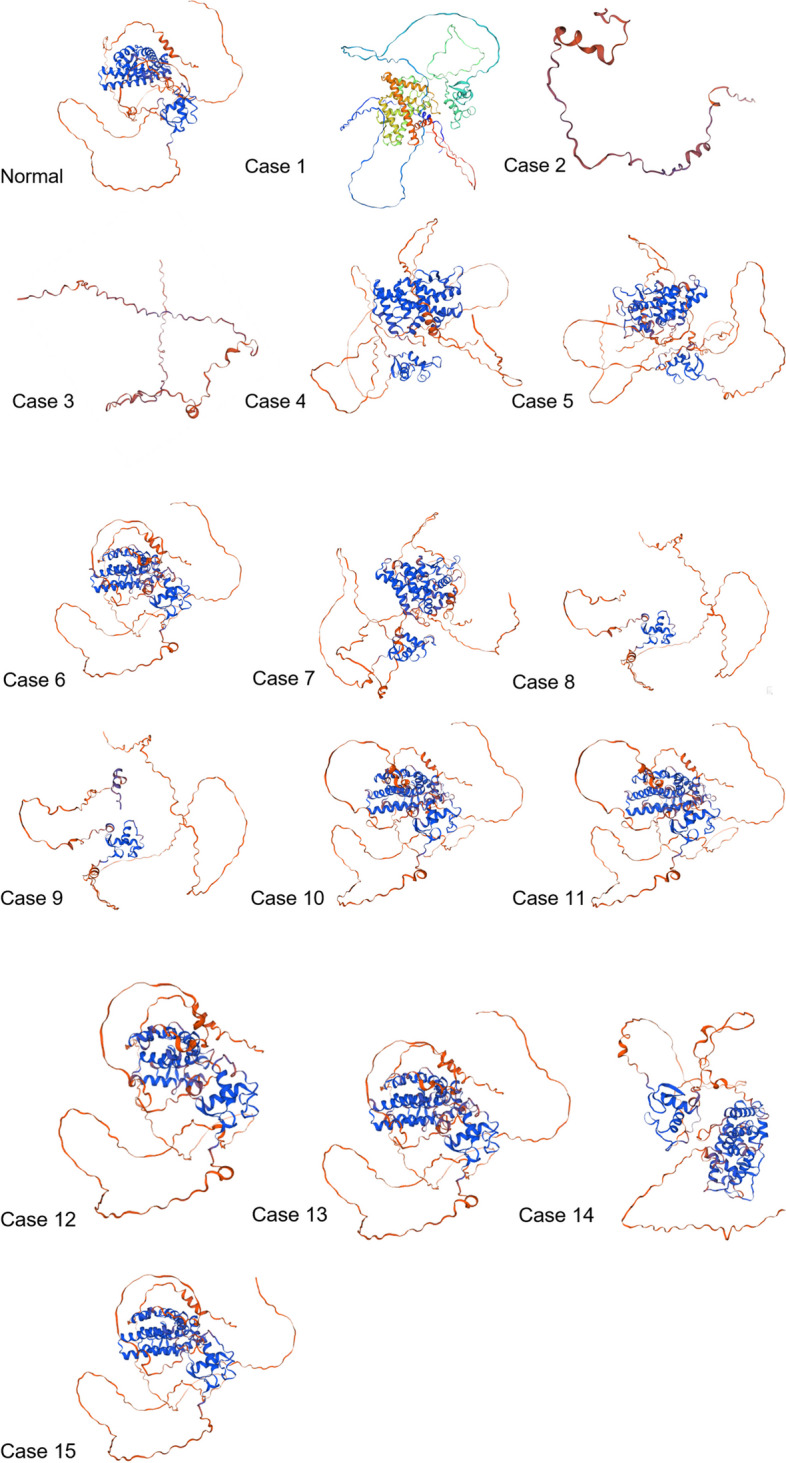
Effect of ESR1 gene SNPs documented in present study on 3D structure of mutated proteins predicted using SWISSMODEL

The SWISSMODEL based analysis of mutated proteins of ESRα gene revealed SNPs rs1554259481, rs188957694 and rs755667747 drastically altered the 3D configuration of mutated proteins. While SNPs rs1583384537 and rs1131692059 caused slight change in structure. However, all other mutations addressed in present study did not affect the overall configuration of mutated proteins (Fig. 2).

**Fig. 3:**
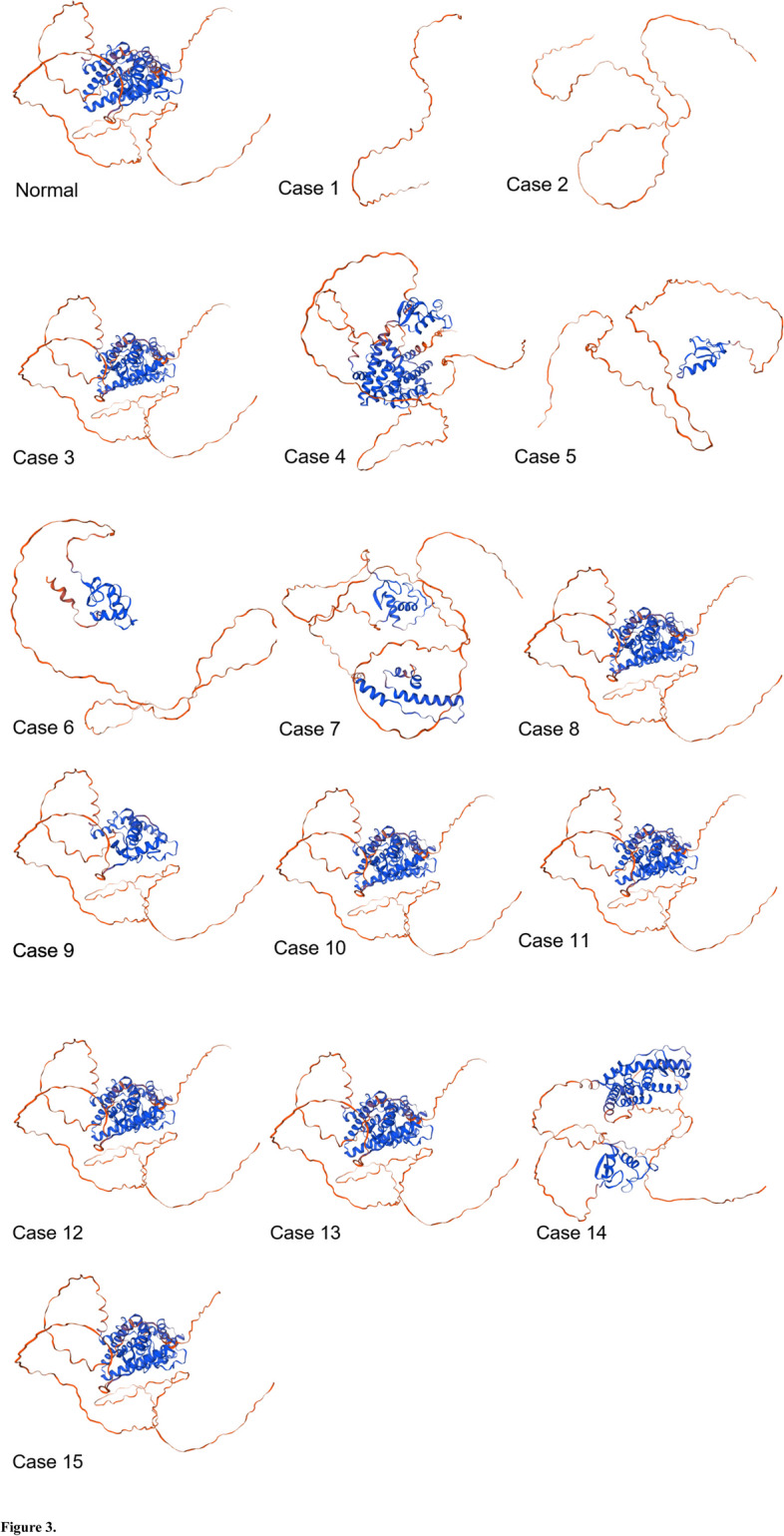
Effect of ESR2 gene SNPs documented in present study on 3D structure of mutated proteins predicted using SWISSMODE

In case of ESRβ gene, mutations rs1463893698, rs140630557, rs1596423459, rs766843910, rs1596405923, rs762454979 and rs1384121511 induced considerable change in mutated proteins. Slight change in 3D structure has been observed in case of SNP rs1257844897. While in all other cases, no effect was observed on tertiary structure of proteins (Fig. 3).

The pdb structures of normal and mutated proteins generated using PHYRE2 tool were subjected to ERRAT and Ramachandran plot analysis. In case of ESRα gene, the values for all the structures including normal and mutated proteins, the ERRAT scores were above 70% and hence, are considered of good quality (Supplementary data Fig. S7). However, in cases of two stop gained SNPs i.e. rs1554259481 and rs104893956 which caused formation of truncated proteins, the overall quality factor was below 50% while in cases of SNPs rs755667747 and rs1467954450, the quality factor was below 70% but above 50%. In ESRβ gene encoded normal and mutated proteins, in all the cases the overall quality factor value was above 70% except rs1596423459, rs140630557, rs766843910 and rs1596405923. For the mutated structure induced by stop-gained mutation rs140630557, no quality score was predicted by ERRAT (Supplementary data Fig. S8).

As far as the validation of protein structures using Ramachandran plots is concerned, the residues found in most favored regions were above or closer to 90% and hence qualified the good quality score. However, in cases of ESRα gene SNPs rs1554259481 and rs104893956 and ESRβ gene SNP rs140630557, the scores were in the range of 70% (Supplementary data Figs. S9 and S10). The G-values were closer to zero in all the cases further validating the protein structures.

### Analysis of SNPs effect on binding tendency of ESR1 and ESR2 receptors with E2

**Fig. 4:**
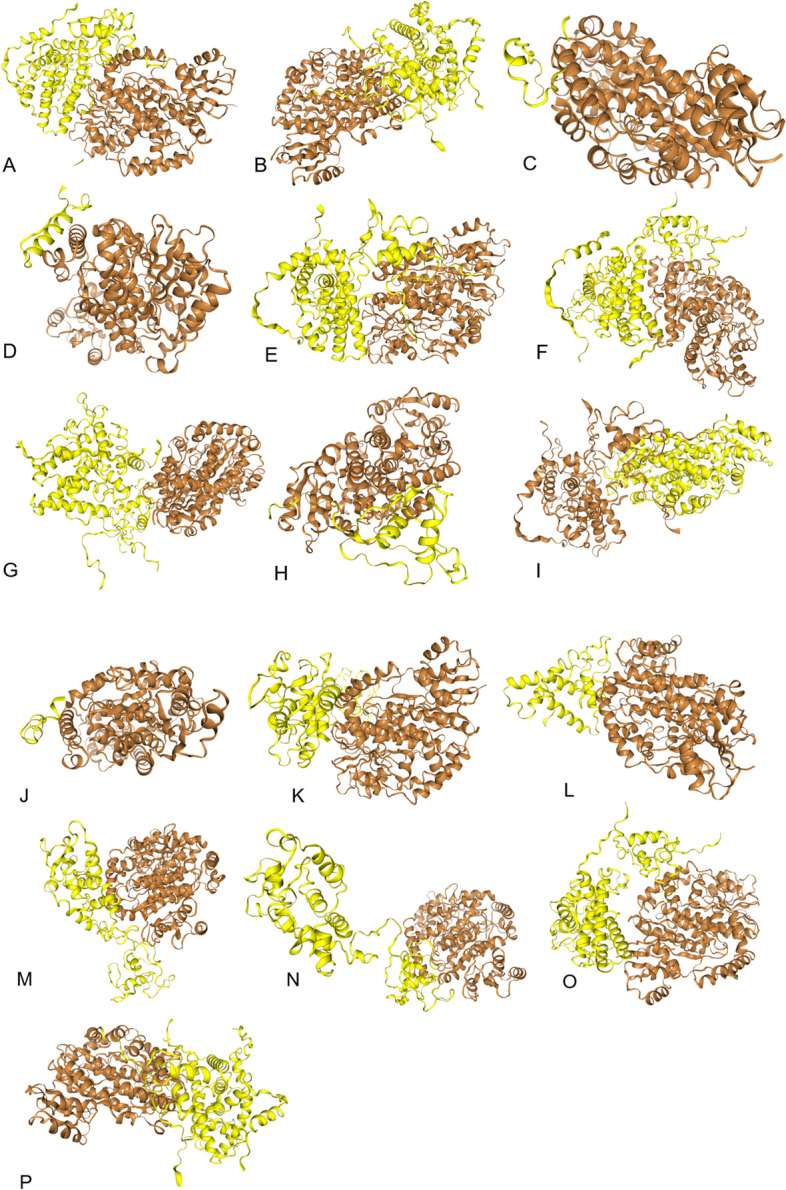
Docking analysis results performed using HDOCK server to predict the effect of SNPs documented in present study on binding tendency of estradiol with ESRα receptor. **a** normal, **b** rs1583384537, **c** rs1554259481, **d** rs104893956, **e** rs761613029, **f** rs778449608, **g** rs866869178, **h** rs188957694, **i** rs755667747, **j** rs1467954450, **k** rs1584799119, **l** rs1131692059, **m** rs762742833, **n** rs758798083, **o** rs1253340312, **p** rs143699938

**Fig. 5:**
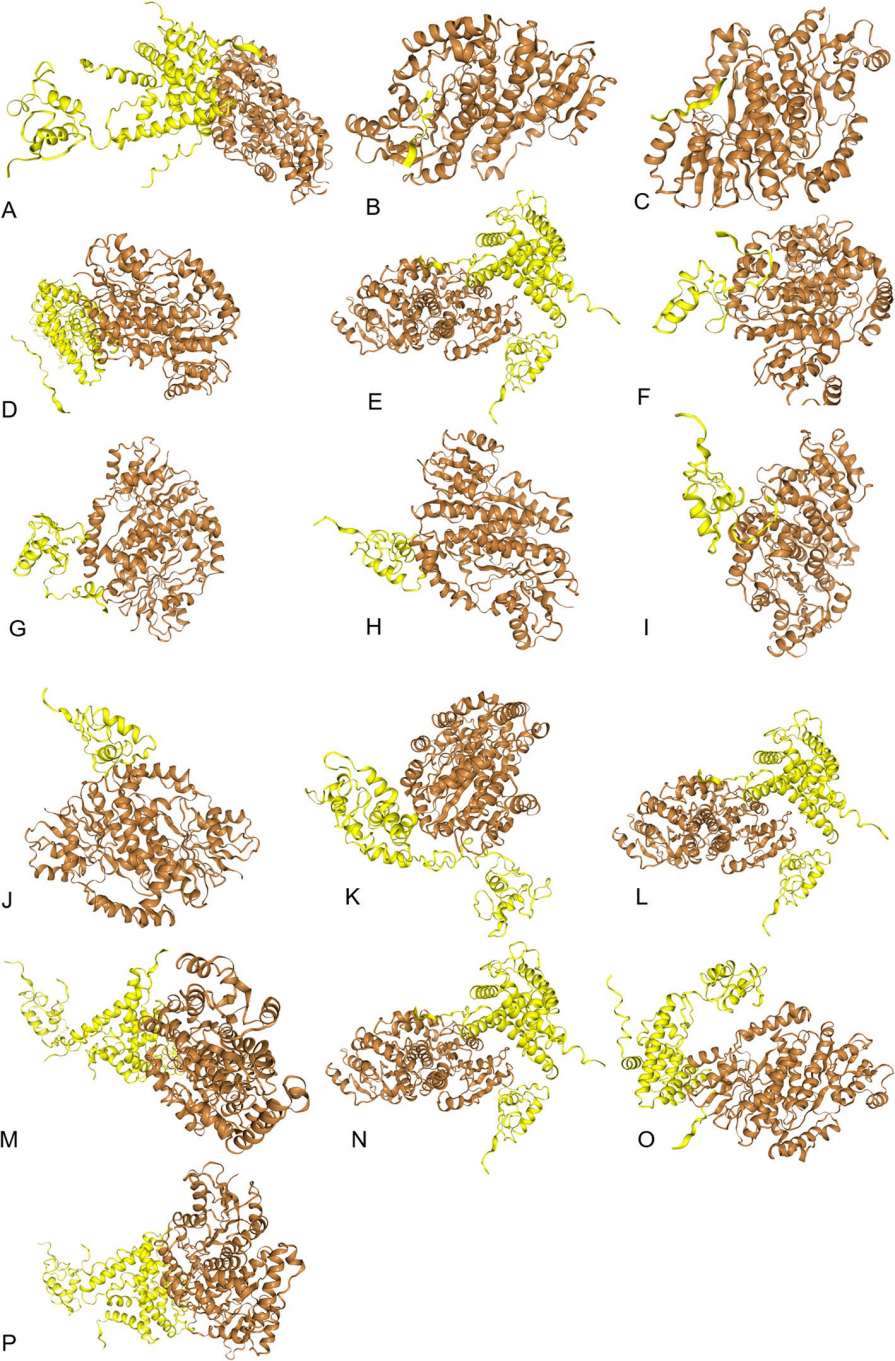
Docking analysis results performed using HDOCK server to predict the effect of SNPs documented in present study on binding tendency of estradiol with ESRβ receptor. **a** normal, **b** rs1463893698, **c** rs140630557, **d** rs1450198518, **e** rs754945292, **f** rs1596423459, **g** rs766843910, **h** rs1596405923, **i** rs762454979, **j** rs1384121511, **k** rs1249242790, **l** rs1414263985, **m** rs78255744, **n** rs768924970, **o** rs1257844897, **p** rs200502775

Docking analysis of mutated forms of ESR1 and ESR2 proteins with E2 depicted highest scores of binding energy in ESR1 cases 1, 5 and 15 documenting SNPs rs1583384537, rs778449608 and rs1436999383, respectively. While in ESR2 cases 3, 4, 5, 6, 7, 9 and 11 – 15 presenting SNPs rs1450198518, rs754945292, rs1596423459, rs766843910, rs1596405923, rs1384121511, rs1414263985, rs78255744, rs768924970, rs1257844897 and rs200502775. Lowest binding tendency was observed in case 9 (rs1467954450) for ESR1 gene and in case 2 (rs140630557) for ESR2 gene (Table 4 and Figs. 4 and 5).

## Discussion

There have been several studies showing the correlation between SNPs of ESR1 and ESR2 genes with PCOS. These studies were based on comparison of genes between the healthy and the diseased individuals using experimental methods including biochemical and hormonal analysis, restriction fragment length polymerase chain reaction (RFLP-PCR), real-time polymerase chain reaction (RT-PCR) and Sanger sequencing [24, 25, 40, 41].

A study focusing at finding *ESR1* and *ESR2 gene* markers associated with PCOS in Tunisian human females revealed strong association of SNVs rs2234693 and rs3798577 in ESRα gene and rs1256049 in ESRβ gene with this disease [40]. Another study has reported the association of SNP rs1999805 in ESR1 gene with PCOS in Chinese population [25]. In Pakistani human females from Punjab, three SNPs i.e. rs2234693, rs9340799 and rs8179176 in ESR1 gene and rs4986938 in *ESR2* gene have been reported to be significantly associated with PCOS [24]. Another research project investigated the correlation between the SNP rs4986938 of the *ESR2* gene with PCOS in unmarried Iraqi human females. This study focused on the substitution of G allele with A allele, with the results indicating the A allele had a higher association with PCOS in comparison to the wild type G allele [42].

Most of the studies available in the literature have focused on biomarkers in intronic and untranslated regions (UTRs) while data about exonic region SNPs is considerably scarce. Moreover, there is lack of detailed information regarding the impact of these mutations on physicochemical characteristics, localization and 2D and 3D structures. The current study aimed to investigate the effects of *ESR1* and *ESR2* genes SNPs reported in Human Genome Project on the attributes of encoded proteins. The SNPs that were focused in present study have not been previously examined in literature.

The pI shows acidity or alkalinity of mutated proteins. In *ESR1* gene, only the mutation rs104893956 enhanced acidity of mutated protein with pI = 4.73 and the mutations rs755667747, rs1467954450 and rs1253340312 had reducing effect on acidity. As far as the ESR2 gene is concerned, the SNP rs1463893698 induced acidity in mutated protein while the mutations rs766843910, rs1596405923, rs762454979 and rs1384121511 increased alkalinity of mutated proteins.

Aliphatic index is the measure of number of amino acids with aliphatic side chain in a protein which reflects protein thermostability over wide range of temperatures. If the value ranges between 66.5 to 84.33 then the protein is considered highly stable [43]. In case of ESR1 gene, only two SNPs i.e. rs755667747 and rs1467954450 decreased thermostability of mutated proteins. The highest thermostability was observed in case of SNP rs1253340312. In case of ESR2 gene, two SNPs i.e. rs1596423459 and rs766843910 reduced the thermostability of mutated proteins while the highest thermostability was induced by mutations rs1257844897 and rs200502775. Instability index indicates stability of protein in test tube [44]. Instability index below 40 reflects protein stability. Among the SNPs documented in *ESR1* gene, only the mutation rs1554259481 has increasing effect on protein stability with instability index of 17.52. As far as the ESR2 gene is concerned, all the mutated proteins just like the normal one were found unstable with instability index above 40. Two SNPs rs1463893698 and rs140630557 reduced stability of mutated proteins to greater extent. GRAVY indicates the polarity level of proteins [45]. In cases of both the ESR1 and ESR2 genes, the negative values of GRAVY indicate that all the mutated proteins are hydrophilic just like the normal one.

In ESR1 gene, three documented SNPs i.e. rs1554259481, rs104893956 and rs755667747 have been found to cause drastic change in physicochemical properties, 2D and 3D structures of mutated proteins. In ESR2 gene, the seven SNPs i.e. rs1463893698, rs140630557, rs1596405923, rs1596423459, rs762454979, rs1384121511 and rs766843910 caused significant alterations in physicochemical properties, 2D and 3D structures of mutant proteins. So, these SNPs can be used as susceptibility markers for PCOS.

Binding of E2 with ESR1 and ESR2 receptors is a crucial event in biological actions of this hormone [46]. Any mutation altering the different attributes of *ESR1* and *ESR2* receptors might lead to disturbance of the binding tendency of E2 with these receptor proteins and might have serious influence on body functions. Therefore, alteration in binding tendency of *ESR1* and *ESR2* proteins mutated forms with E2 has been observed in present study. Single nucleotide variants i.e. rs1467954450 (ESR1 receptor), rs1463893698 and rs140630557 (ESR2 receptor) markedly reduced this binding as revealed by their docking scores. Although literature reports the association of ESR1 gene heterozygous mutation c. 619G > A/p.A207T with insensitivity of the encoded receptor towards E2 as well as the association of ESR genes polymorphisms with disturbances in E2 concentration in PCOS patients [21, 47]. However, this is the first ever study reporting the effect of rs1467954450 (ESR1 receptor), rs1463893698 and rs140630557 SNPs in ESRα and ESRβ genes on binding with E2. This reduced binding affinity might also contribute to disturbance in effective level of E2 hormone in the body.

## Conclusion

The current *in-silico* study has demonstrated a strong association between ten SNPs present in *ESR1* and *ESR2* genes with PCOS. However, it is vital to conduct further research to assess the potential of these mutations as PCOS biomarkers. Identifying these SNPs could assist in predicting the likelihood of PCOS development in human females. These findings could also contribute to the development of targeted therapies for PCOS and help improve the understanding of the underlying molecular mechanisms involved in the pathogenesis of the disorder.

### Supplementary Information

**Additional file 1:** **Supplementary data Figure S1.** Analysis of effect of SNPs in ESR1 gene on sub-cellular localization of mutated proteins. **Supplementary data Figure S2.** Analysis of effect of SNPs in ESR2 gene on sub-cellular localization of mutated proteins. **Supplementary data Figure S3.** Sequences of normal and mutated proteins encoded by ESR1 gene, the disordered regions are represented in red color. **Supplementary data Figure S4.** Sequences of normal and mutated proteins encoded by ESR2 gene, the disordered regions are represented in red color. **Supplementary data Figure S5.** Disorder profile plots for normal and the mutated ESRα proteins analyzed in present study based on PrDOS tool. **Supplementary data Figure S6.** Disorder profile plots for normal and the mutated ESRβ proteins analyzed in present study based on PrDOS tool. **Supplementary data Figure S7.** Validation of PHYRE2 tool generated pdb structures of normal and mutated proteins encoded by ESR1 gene using ERRAT2 tool. **Supplementary data Figure S8.** Validation of PHYRE2 tool generated pdb structures of normal and mutated proteins encoded by ESR2 gene using ERRAT2 tool. **Supplementary data Figure S9.** Validation of PHYRE2 tool generated pdb structures of normal and mutated proteins encoded by ESR1 gene using Ramachandran plots. **Supplementary data Figure S10.** Validation of PHYRE2 tool generated pdb structures of normal and mutated proteins encoded by ESR2 gene using Ramachandran plots. **Supplementary data Table 1.** Transcript IDs and CDS of ESRα and ESRβ genes analyzed in present study. **Supplementary data Table 2.** Predicting the effect of SNPs on number of disordered regions and the number of disordered amino acids of ESRα and ESRβ genes predicted using PrDOS server.

## Data Availability

SNPs documented in present study have been retrieved form ENSEMBL database (https://web.expasy.org/translate).

## Abbreviations

PCOS: Polycystic Ovary Syndrome
CYP11A1: Cytochrome P450, family 11, sub-family A, member 1
CYP17: Cytochrome P450, family 17
CYP19: Cytochrome P450, family 19
CYP21: Cytochrome P450, family 21
AR: Androgen receptor gene
SHBG: Sex hormone binding globular protein
LH: Luteinizing hormone
FSHR: Follicle stimulating hormone receptor
AMH: Anti-mullerian hormone
CAPN10: Calpain 10
IRS-1: Insulin receptor substrate-1
IRS-2: Insulin receptor substrate-2
INS: Insulin
EEDs: Environmental endocrine disruptors
SIFT: Sorting Intolerant from Tolerant
PolyPhen: Polymorphism Phenotyping
CADD: Combined Annotation Dependent Depletion
REVEL: Rare Exome Variant Ensemble Learner
MetaLR: Meta Logistic Regression
DGG: Gibbs free energy
RI: Reliability index
GRAVY: Grand average of hydropathicity
pI: Isoelectric point
PrDOS: Protein disorder prediction server
RFLP-PCR: Restriction fragment length polymerase chain reaction
RT-PCR: Real-time polymerase chain reaction
UTRs: Untranslated regions
