# Supplementary material for: Exploring the association of ESR1 and ESR2 gene SNPs with polycystic ovary syndrome in human females: a comprehensive association study
Source: J Ovarian Res. 2024 Jan 29;17:27. doi: 10.1186/s13048-023-01335-7 (PMC10823698; doi:10.1186/s13048-023-01335-7)
Supplement: Supplementary file 1 — Additional file 1: Supplementary data Figure S1. Analysis of effect of SNPs in ESR1 gene on sub-cellular localization of mutated proteins. Supplementary data Figure S2. Analysis of effect of SNPs in ESR2 gene on sub-cellular localization of mutated proteins. Supplementary data Figure S3. Sequences of normal and mutated proteins encoded by ESR1 gene, the disordered regions are represented in red color. Supplementary data Figure S4. Sequences of normal and mutated proteins encoded by ESR2 gene, the disordered regions are represented in red color. Supplementary data Figure S5. Disorder profile plots for normal and the mutated ESRα proteins analyzed in present study based on PrDOS tool. Supplementary data Figure S6. Disorder profile plots for normal and the mutated ESRβ proteins analyzed in present study based on PrDOS tool. Supplementary data Figure S7. Validation of PHYRE2 tool generated pdb structures of normal and mutated proteins encoded by ESR1 gene using ERRAT2 tool. Supplementary data Figure S8. Validation of PHYRE2 tool generated pdb structures of normal and mutated proteins encoded by ESR2 gene using ERRAT2 tool. Supplementary data Figure S9. Validation of PHYRE2 tool generated pdb structures of normal and mutated proteins encoded by ESR1 gene using Ramachandran plots. Supplementary data Figure S10. Validation of PHYRE2 tool generated pdb structures of normal and mutated proteins encoded by ESR2 gene using Ramachandran plots. Supplementary data Table 1. Transcript IDs and CDS of ESRα and ESRβ genes analyzed in present study. Supplementary data Table 2. Predicting the effect of SNPs on number of disordered regions and the number of disordered amino acids of ESRα and ESRβ genes predicted using PrDOS server. [file 13048_2023_1335_MOESM1_ESM.docx]

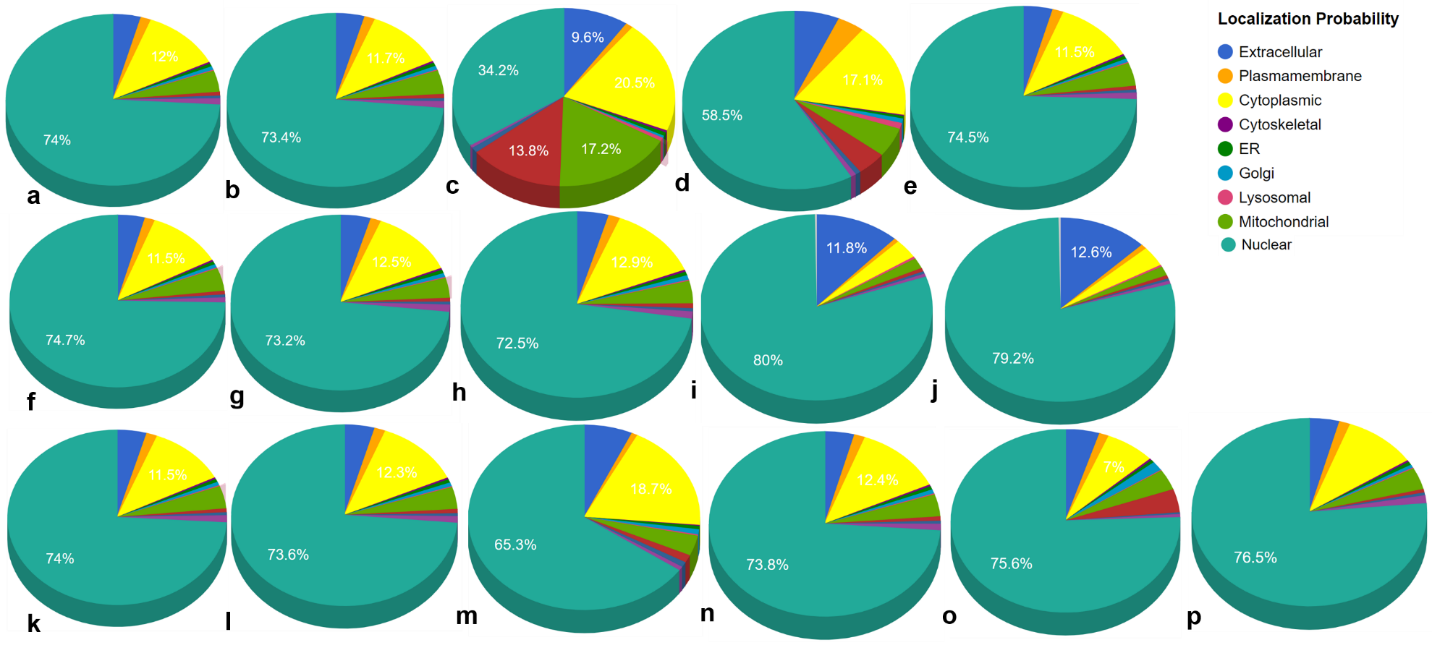


**Supplementary data Figure S1:** Analysis of effect of SNPs in ESR1 gene on sub-cellular localization of mutated proteins

(a) normal, (b) [rs1583384537](https://asia.ensembl.org/Homo_sapiens/Variation/Summary?db=core;g=ENSG00000091831;r=6:151656691-152129619;t=ENST00000206249;vf=690836708), (c) [rs1554259481](https://asia.ensembl.org/Homo_sapiens/Variation/Summary?db=core;g=ENSG00000091831;r=6:151656691-152129619;t=ENST00000206249;vf=672757261), (d) [rs104893956](https://asia.ensembl.org/Homo_sapiens/Variation/Summary?db=core;g=ENSG00000091831;r=6:151656691-152129619;t=ENST00000206249;vf=186352285), (e) [rs761613029](https://asia.ensembl.org/Homo_sapiens/Variation/Summary?db=core;g=ENSG00000091831;r=6:151656691-152129619;t=ENST00000206249;vf=301776079), (f) [rs778449608](https://asia.ensembl.org/Homo_sapiens/Variation/Summary?db=core;g=ENSG00000091831;r=6:151656691-152129619;t=ENST00000206249;vf=314669151), (g) [rs866869178](https://asia.ensembl.org/Homo_sapiens/Variation/Summary?db=core;g=ENSG00000091831;r=6:151656691-152129619;t=ENST00000206249;vf=317238920), (h) [rs188957694](https://asia.ensembl.org/Homo_sapiens/Variation/Summary?db=core;g=ENSG00000091831;r=6:151656691-152129619;t=ENST00000206249;vf=213279813), (i) [rs755667747](https://asia.ensembl.org/Homo_sapiens/Variation/Summary?db=core;g=ENSG00000091831;r=6:151656691-152129619;t=ENST00000206249;vf=298272399), (j) [rs1467954450](https://asia.ensembl.org/Homo_sapiens/Variation/Summary?db=core;g=ENSG00000091831;r=6:151656691-152129619;t=ENST00000206249;vf=664784020), (k) [rs1584799119](https://asia.ensembl.org/Homo_sapiens/Variation/Summary?db=core;g=ENSG00000091831;r=6:151656691-152129619;t=ENST00000206249;vf=692267559), (l) [rs1131692059](https://asia.ensembl.org/Homo_sapiens/Variation/Summary?db=core;g=ENSG00000091831;r=6:151656691-152129619;t=ENST00000206249;vf=525229385), (m) [rs762742833](https://asia.ensembl.org/Homo_sapiens/Variation/Summary?db=core;g=ENSG00000091831;r=6:151656691-152129619;t=ENST00000206249;vf=302413577), (n) [rs758798083](https://asia.ensembl.org/Homo_sapiens/Variation/Summary?db=core;g=ENSG00000091831;r=6:151656691-152129619;t=ENST00000206249;vf=300171136), (o) [rs1253340312](https://asia.ensembl.org/Homo_sapiens/Variation/Summary?db=core;g=ENSG00000091831;r=6:151656691-152129619;t=ENST00000206249;vf=580549169), (p) [rs1436999383](https://asia.ensembl.org/Homo_sapiens/Variation/Summary?db=core;g=ENSG00000091831;r=6:151656691-152129619;t=ENST00000206249;vf=654831386)


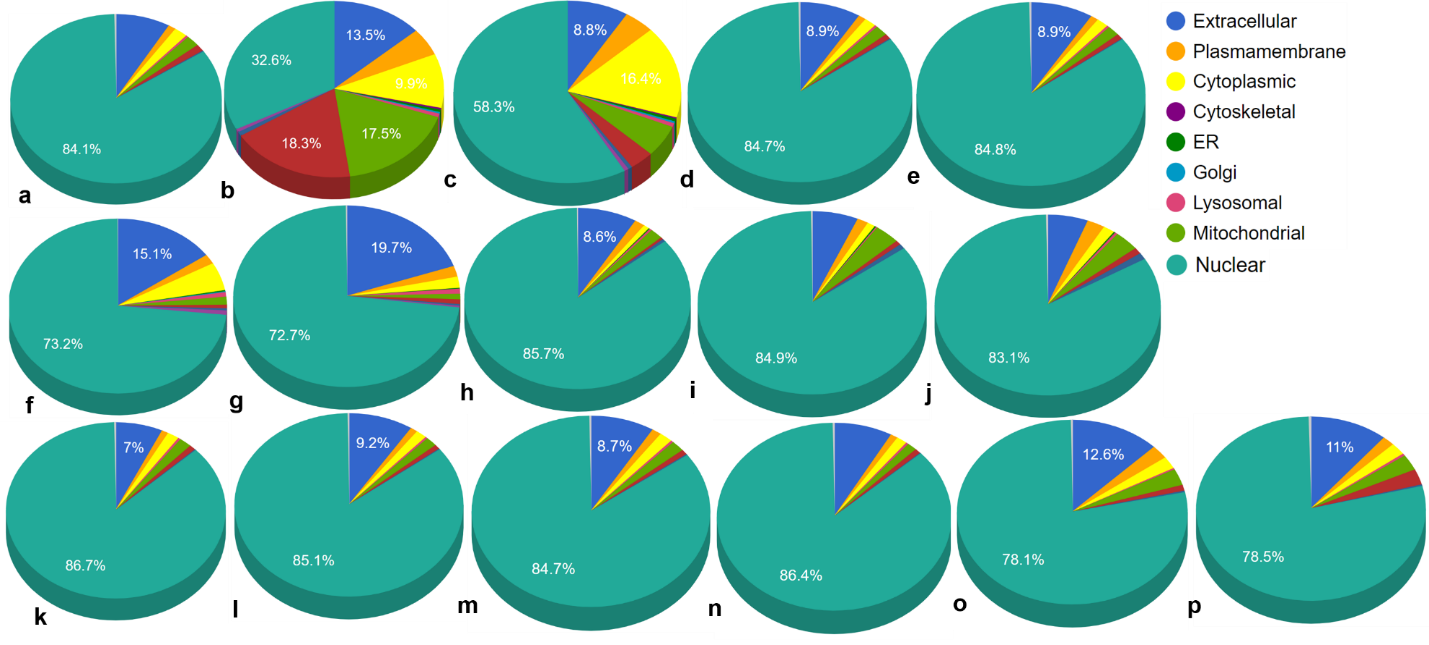


**Supplementary data Figure S2:** Analysis of effect of SNPs in ESR2 gene on sub-cellular localization of mutated proteins

(a) normal , (b) [rs1463893698](https://asia.ensembl.org/Homo_sapiens/Variation/Summary?db=core;g=ENSG00000140009;r=14:64084232-64338112;t=ENST00000341099;vf=487477622), (c) [rs140630557](https://asia.ensembl.org/Homo_sapiens/Variation/Summary?db=core;g=ENSG00000140009;r=14:64084232-64338112;t=ENST00000341099;vf=187325610), (d) [rs1450198518](https://asia.ensembl.org/Homo_sapiens/Variation/Summary?db=core;g=ENSG00000140009;r=14:64084232-64338112;t=ENST00000341099;vf=481112348), (e) [rs754945292](https://asia.ensembl.org/Homo_sapiens/Variation/Summary?db=core;g=ENSG00000140009;r=14:64084232-64338112;t=ENST00000341099;vf=252034067), (f) [rs1596423459](https://asia.ensembl.org/Homo_sapiens/Variation/Summary?db=core;g=ENSG00000140009;r=14:64084232-64338112;t=ENST00000341099;vf=523934685), (g) [rs766843910](https://asia.ensembl.org/Homo_sapiens/Variation/Summary?db=core;g=ENSG00000140009;r=14:64084232-64338112;t=ENST00000341099;vf=264787059), (h) [rs1596405923](https://asia.ensembl.org/Homo_sapiens/Variation/Summary?db=core;g=ENSG00000140009;r=14:64084232-64338112;t=ENST00000341099;vf=523929087), (i) [rs762454979](https://asia.ensembl.org/Homo_sapiens/Variation/Summary?db=core;g=ENSG00000140009;r=14:64084232-64338112;t=ENST00000341099;vf=254094829), (j) [rs1384121511](https://asia.ensembl.org/Homo_sapiens/Variation/Summary?db=core;g=ENSG00000140009;r=14:64084232-64338112;t=ENST00000341099;vf=459145327), (k) [rs1249242790](https://asia.ensembl.org/Homo_sapiens/Variation/Summary?db=core;g=ENSG00000140009;r=14:64084232-64338112;t=ENST00000341099;vf=419316481), (l) [rs1414263985](https://asia.ensembl.org/Homo_sapiens/Variation/Summary?db=core;g=ENSG00000140009;r=14:64084232-64338112;t=ENST00000341099;vf=469984932), (m) [rs78255744](https://asia.ensembl.org/Homo_sapiens/Variation/Summary?db=core;g=ENSG00000140009;r=14:64084232-64338112;t=ENST00000341099;vf=184777125), (n) [rs768924970](https://asia.ensembl.org/Homo_sapiens/Variation/Summary?db=core;g=ENSG00000140009;r=14:64084232-64338112;t=ENST00000341099;vf=265336834), (o) [rs1257844897](https://asia.ensembl.org/Homo_sapiens/Variation/Summary?db=core;g=ENSG00000140009;r=14:64084232-64338112;t=ENST00000341099;vf=428748888), (p) [rs200502775](https://asia.ensembl.org/Homo_sapiens/Variation/Summary?db=core;g=ENSG00000140009;r=14:64084232-64338112;t=ENST00000341099;vf=197621052)


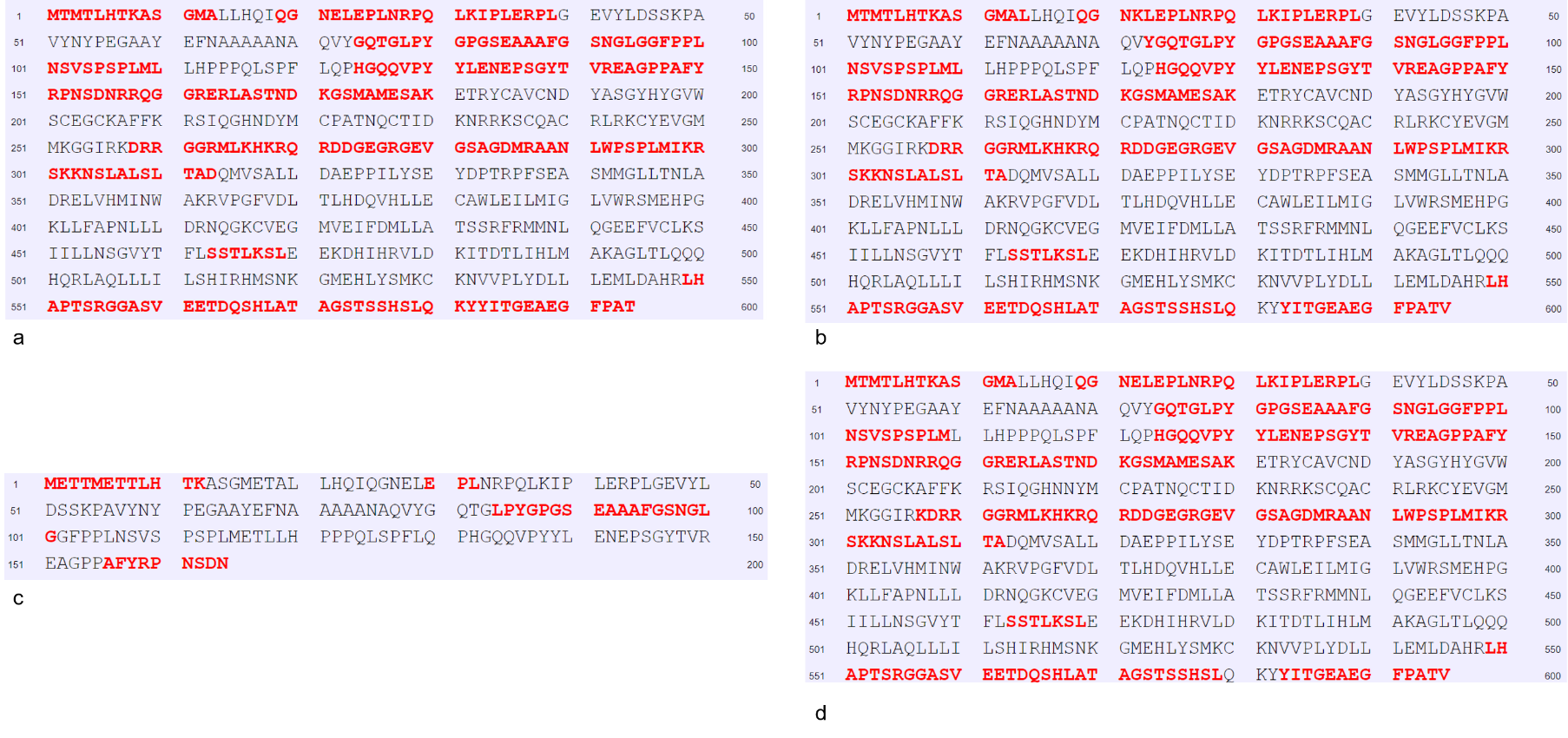


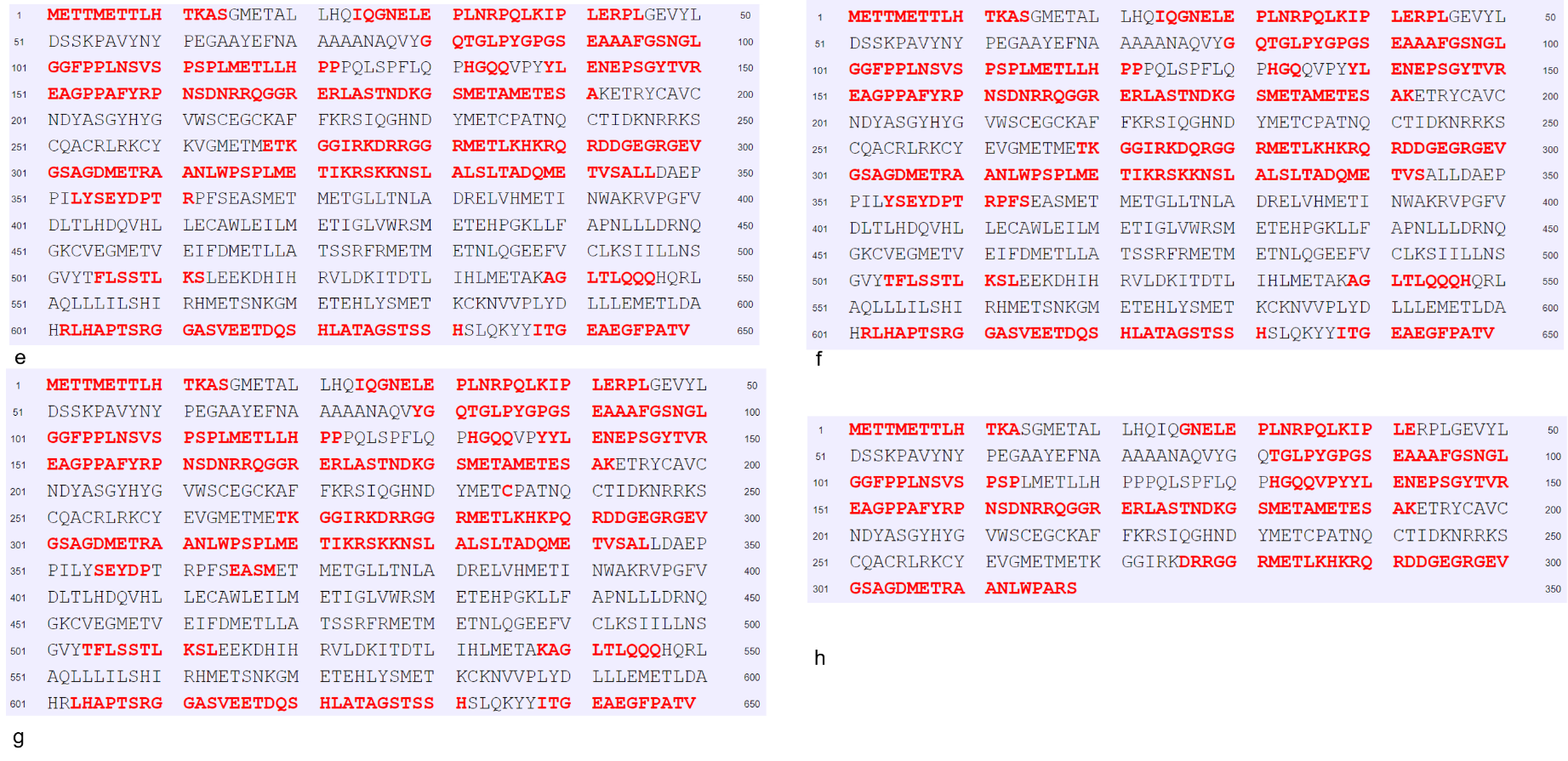


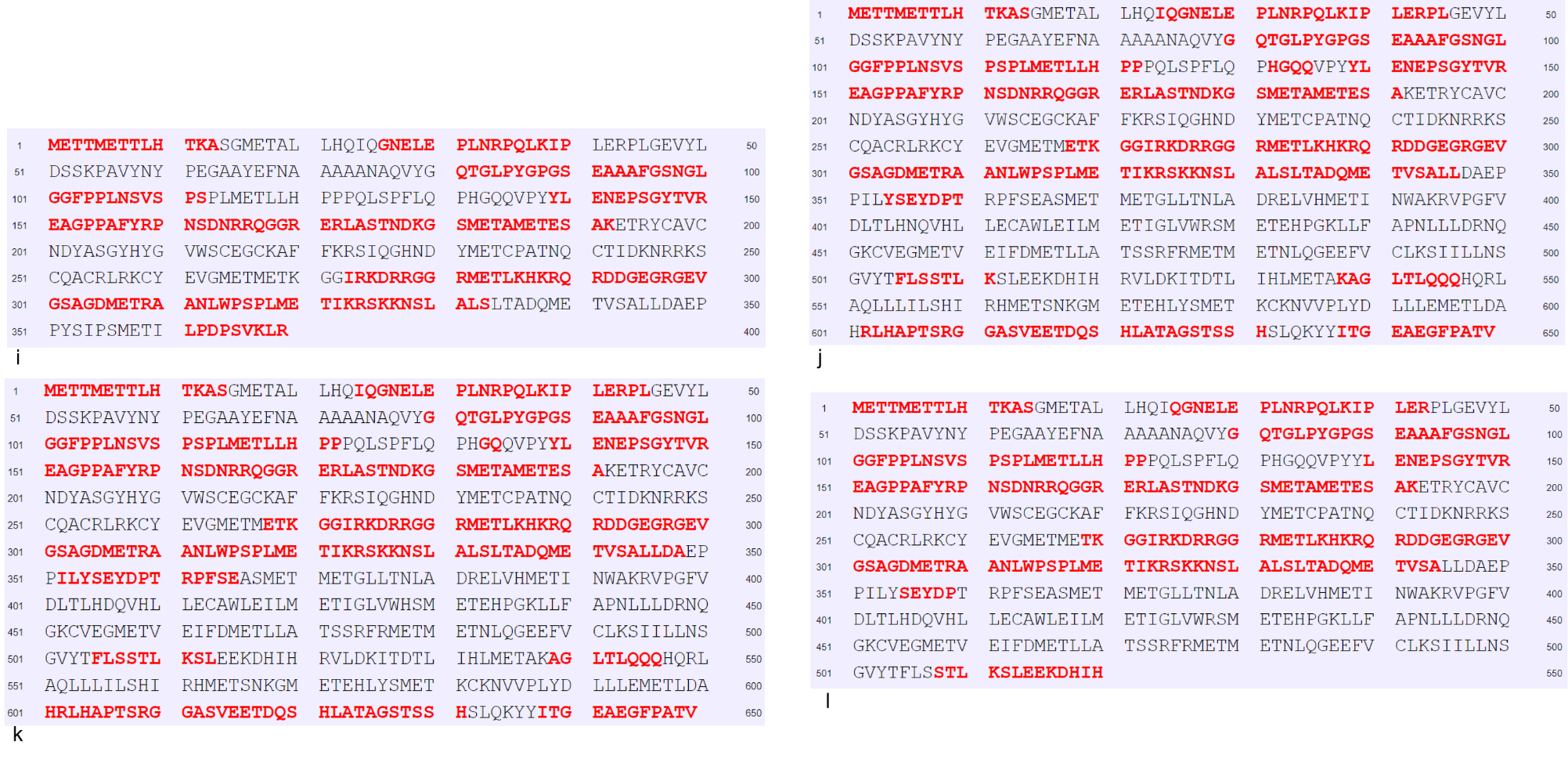


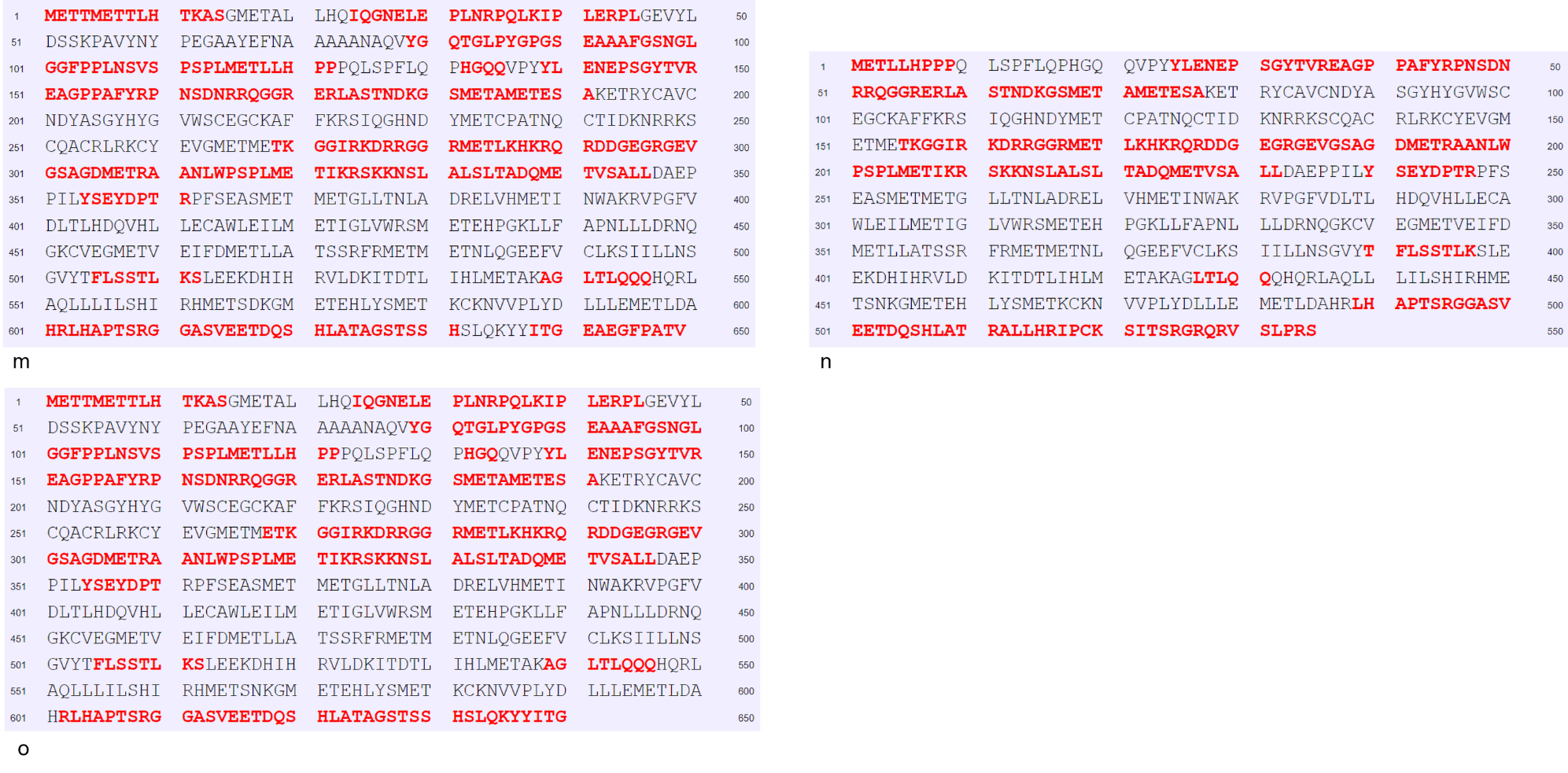


**Supplementary data Figure S3:** Sequences of normal and mutated proteins encoded by ESR1 gene, the disordered regions are represented in red color

(a) normal, (b) [rs1583384537](https://asia.ensembl.org/Homo_sapiens/Variation/Summary?db=core;g=ENSG00000091831;r=6:151656691-152129619;t=ENST00000206249;vf=690836708), (c) [rs1554259481](https://asia.ensembl.org/Homo_sapiens/Variation/Summary?db=core;g=ENSG00000091831;r=6:151656691-152129619;t=ENST00000206249;vf=672757261), (d) [rs104893956](https://asia.ensembl.org/Homo_sapiens/Variation/Summary?db=core;g=ENSG00000091831;r=6:151656691-152129619;t=ENST00000206249;vf=186352285), (e) [rs761613029](https://asia.ensembl.org/Homo_sapiens/Variation/Summary?db=core;g=ENSG00000091831;r=6:151656691-152129619;t=ENST00000206249;vf=301776079), (f) [rs778449608](https://asia.ensembl.org/Homo_sapiens/Variation/Summary?db=core;g=ENSG00000091831;r=6:151656691-152129619;t=ENST00000206249;vf=314669151), (g) [rs866869178](https://asia.ensembl.org/Homo_sapiens/Variation/Summary?db=core;g=ENSG00000091831;r=6:151656691-152129619;t=ENST00000206249;vf=317238920), (h) [rs188957694](https://asia.ensembl.org/Homo_sapiens/Variation/Summary?db=core;g=ENSG00000091831;r=6:151656691-152129619;t=ENST00000206249;vf=213279813), (i) [rs755667747](https://asia.ensembl.org/Homo_sapiens/Variation/Summary?db=core;g=ENSG00000091831;r=6:151656691-152129619;t=ENST00000206249;vf=298272399), (j) [rs1467954450](https://asia.ensembl.org/Homo_sapiens/Variation/Summary?db=core;g=ENSG00000091831;r=6:151656691-152129619;t=ENST00000206249;vf=664784020), (k) [rs1584799119](https://asia.ensembl.org/Homo_sapiens/Variation/Summary?db=core;g=ENSG00000091831;r=6:151656691-152129619;t=ENST00000206249;vf=692267559), (l) [rs1131692059](https://asia.ensembl.org/Homo_sapiens/Variation/Summary?db=core;g=ENSG00000091831;r=6:151656691-152129619;t=ENST00000206249;vf=525229385), (m) [rs762742833](https://asia.ensembl.org/Homo_sapiens/Variation/Summary?db=core;g=ENSG00000091831;r=6:151656691-152129619;t=ENST00000206249;vf=302413577), (n) [rs758798083](https://asia.ensembl.org/Homo_sapiens/Variation/Summary?db=core;g=ENSG00000091831;r=6:151656691-152129619;t=ENST00000206249;vf=300171136), (o) [rs1253340312](https://asia.ensembl.org/Homo_sapiens/Variation/Summary?db=core;g=ENSG00000091831;r=6:151656691-152129619;t=ENST00000206249;vf=580549169), (p) rs143699938


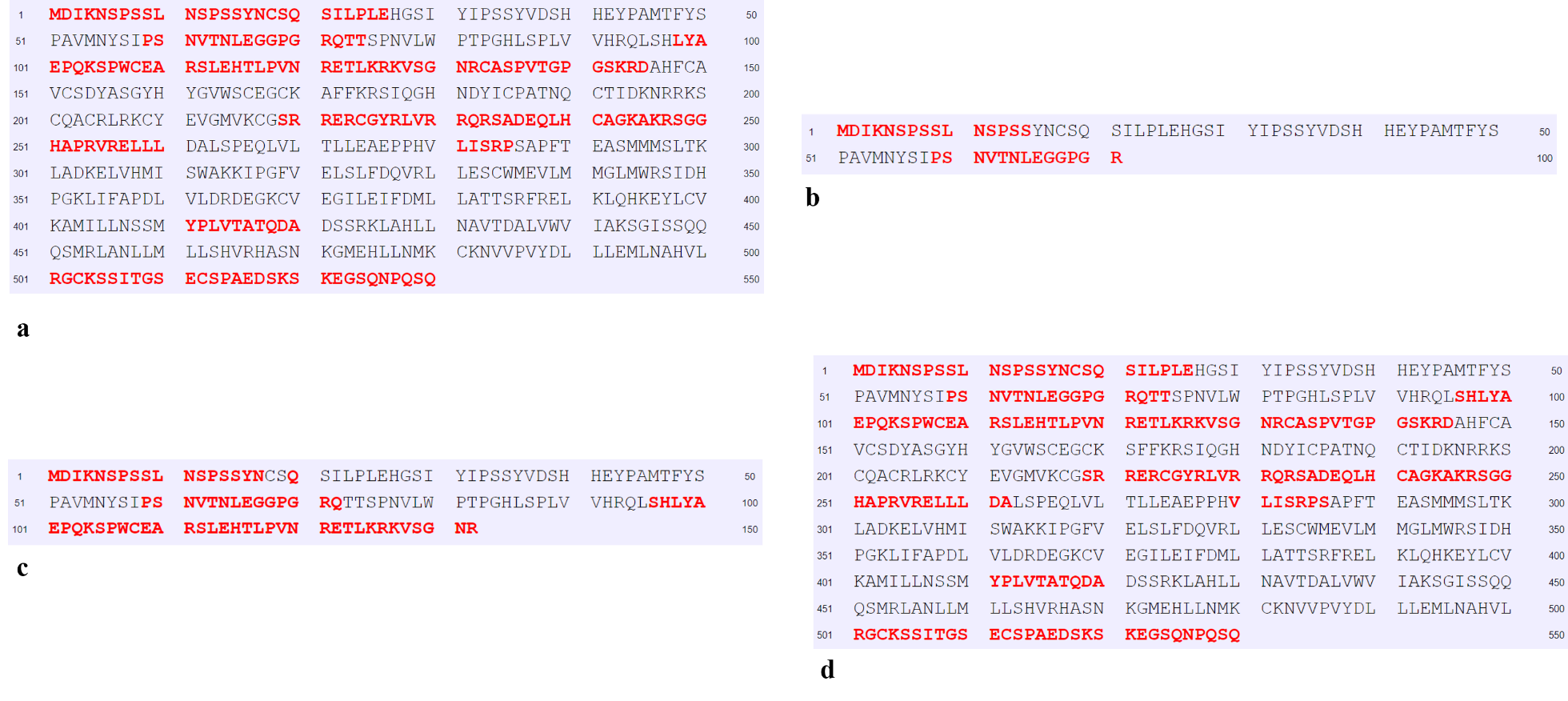


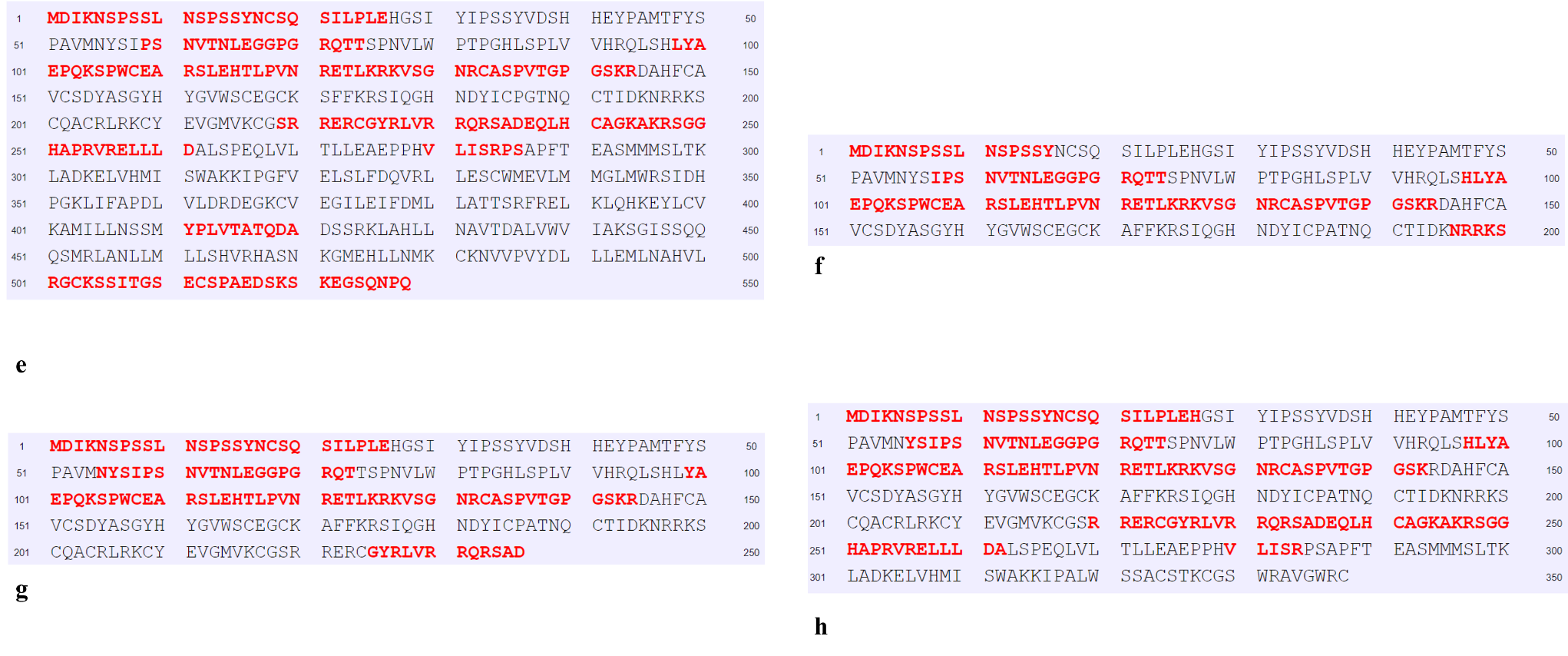


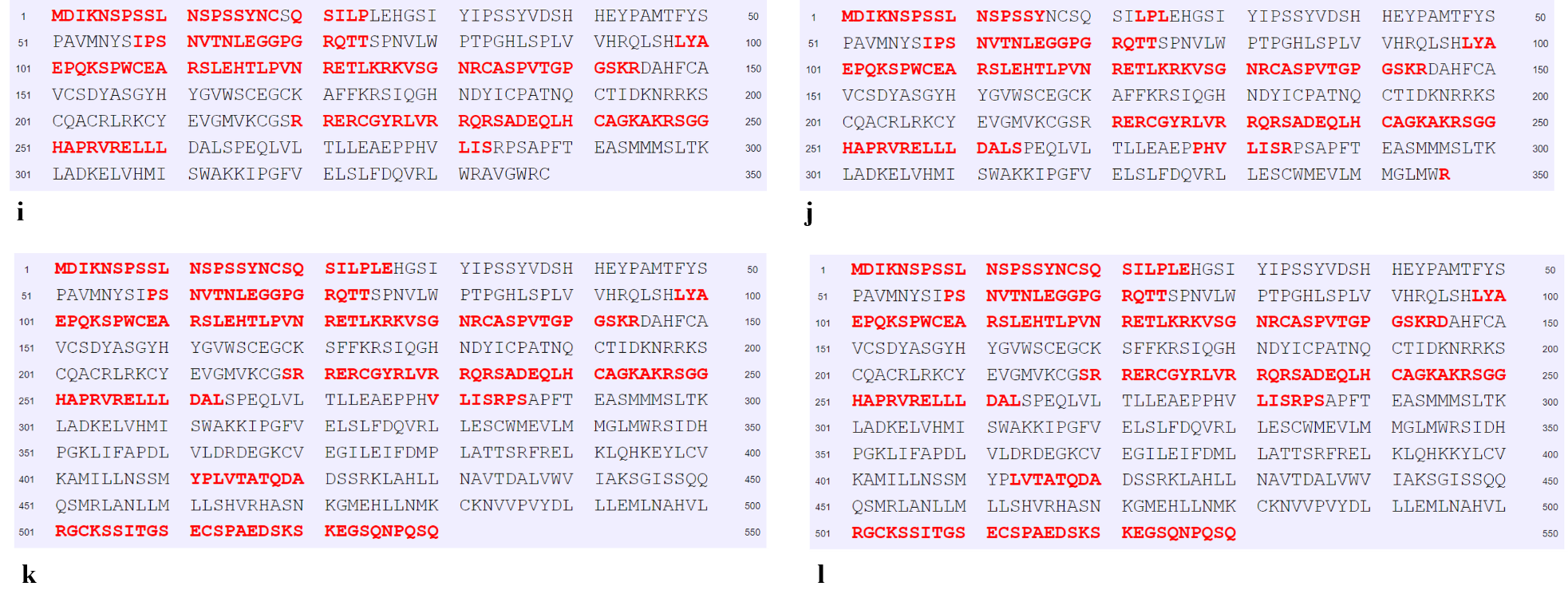


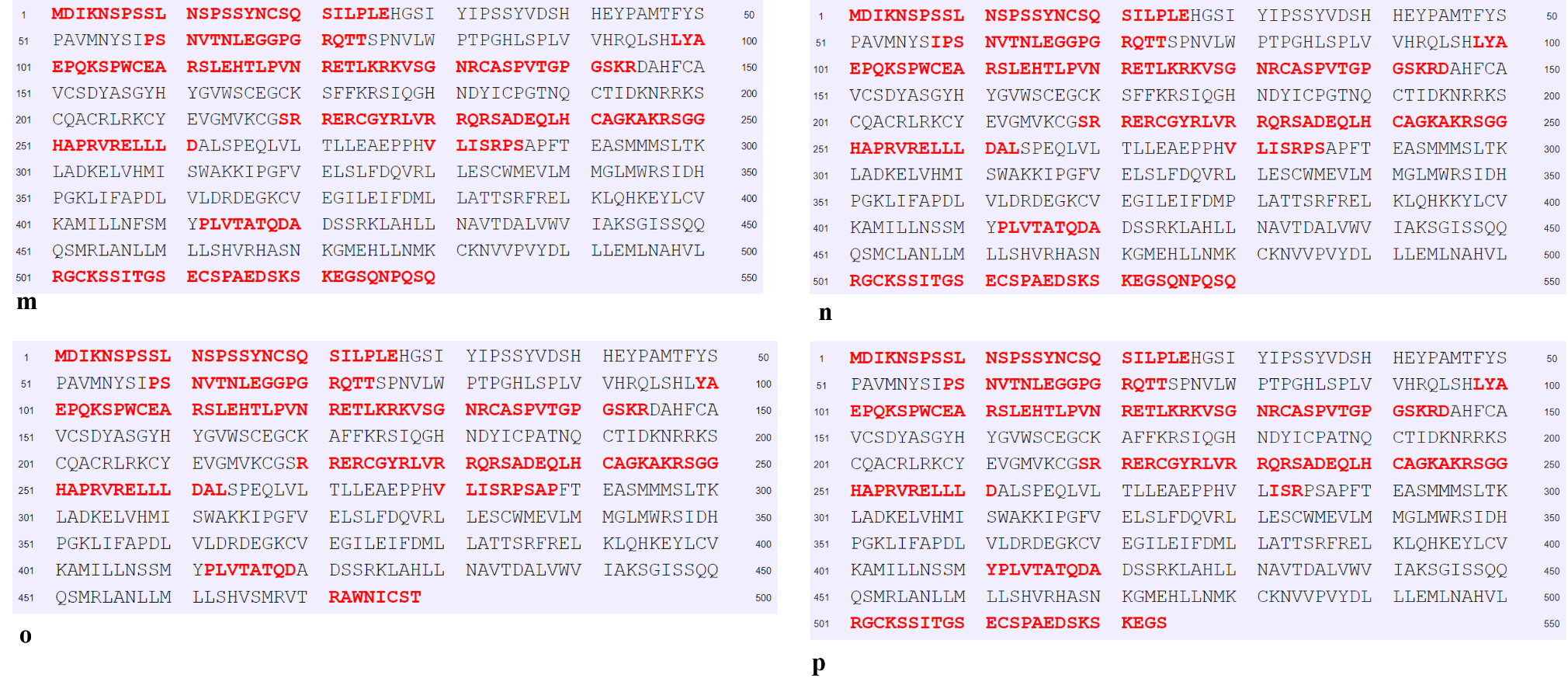


**Supplementary data Figure S4:** Sequences of normal and mutated proteins encoded by ESR2 gene, the disordered regions are represented in red color

(a) normal , (b) [rs1463893698](https://asia.ensembl.org/Homo_sapiens/Variation/Summary?db=core;g=ENSG00000140009;r=14:64084232-64338112;t=ENST00000341099;vf=487477622), (c) [rs140630557](https://asia.ensembl.org/Homo_sapiens/Variation/Summary?db=core;g=ENSG00000140009;r=14:64084232-64338112;t=ENST00000341099;vf=187325610), (d) [rs1450198518](https://asia.ensembl.org/Homo_sapiens/Variation/Summary?db=core;g=ENSG00000140009;r=14:64084232-64338112;t=ENST00000341099;vf=481112348), (e) [rs754945292](https://asia.ensembl.org/Homo_sapiens/Variation/Summary?db=core;g=ENSG00000140009;r=14:64084232-64338112;t=ENST00000341099;vf=252034067), (f) [rs1596423459](https://asia.ensembl.org/Homo_sapiens/Variation/Summary?db=core;g=ENSG00000140009;r=14:64084232-64338112;t=ENST00000341099;vf=523934685), (g) [rs766843910](https://asia.ensembl.org/Homo_sapiens/Variation/Summary?db=core;g=ENSG00000140009;r=14:64084232-64338112;t=ENST00000341099;vf=264787059), (h) [rs1596405923](https://asia.ensembl.org/Homo_sapiens/Variation/Summary?db=core;g=ENSG00000140009;r=14:64084232-64338112;t=ENST00000341099;vf=523929087), (i) [rs762454979](https://asia.ensembl.org/Homo_sapiens/Variation/Summary?db=core;g=ENSG00000140009;r=14:64084232-64338112;t=ENST00000341099;vf=254094829), (j) [rs1384121511](https://asia.ensembl.org/Homo_sapiens/Variation/Summary?db=core;g=ENSG00000140009;r=14:64084232-64338112;t=ENST00000341099;vf=459145327), (k) [rs1249242790](https://asia.ensembl.org/Homo_sapiens/Variation/Summary?db=core;g=ENSG00000140009;r=14:64084232-64338112;t=ENST00000341099;vf=419316481), (l) [rs1414263985](https://asia.ensembl.org/Homo_sapiens/Variation/Summary?db=core;g=ENSG00000140009;r=14:64084232-64338112;t=ENST00000341099;vf=469984932), (m) [rs78255744](https://asia.ensembl.org/Homo_sapiens/Variation/Summary?db=core;g=ENSG00000140009;r=14:64084232-64338112;t=ENST00000341099;vf=184777125), (n) [rs768924970](https://asia.ensembl.org/Homo_sapiens/Variation/Summary?db=core;g=ENSG00000140009;r=14:64084232-64338112;t=ENST00000341099;vf=265336834), (o) [rs1257844897](https://asia.ensembl.org/Homo_sapiens/Variation/Summary?db=core;g=ENSG00000140009;r=14:64084232-64338112;t=ENST00000341099;vf=428748888), (p) [rs200502775](https://asia.ensembl.org/Homo_sapiens/Variation/Summary?db=core;g=ENSG00000140009;r=14:64084232-64338112;t=ENST00000341099;vf=197621052)

**
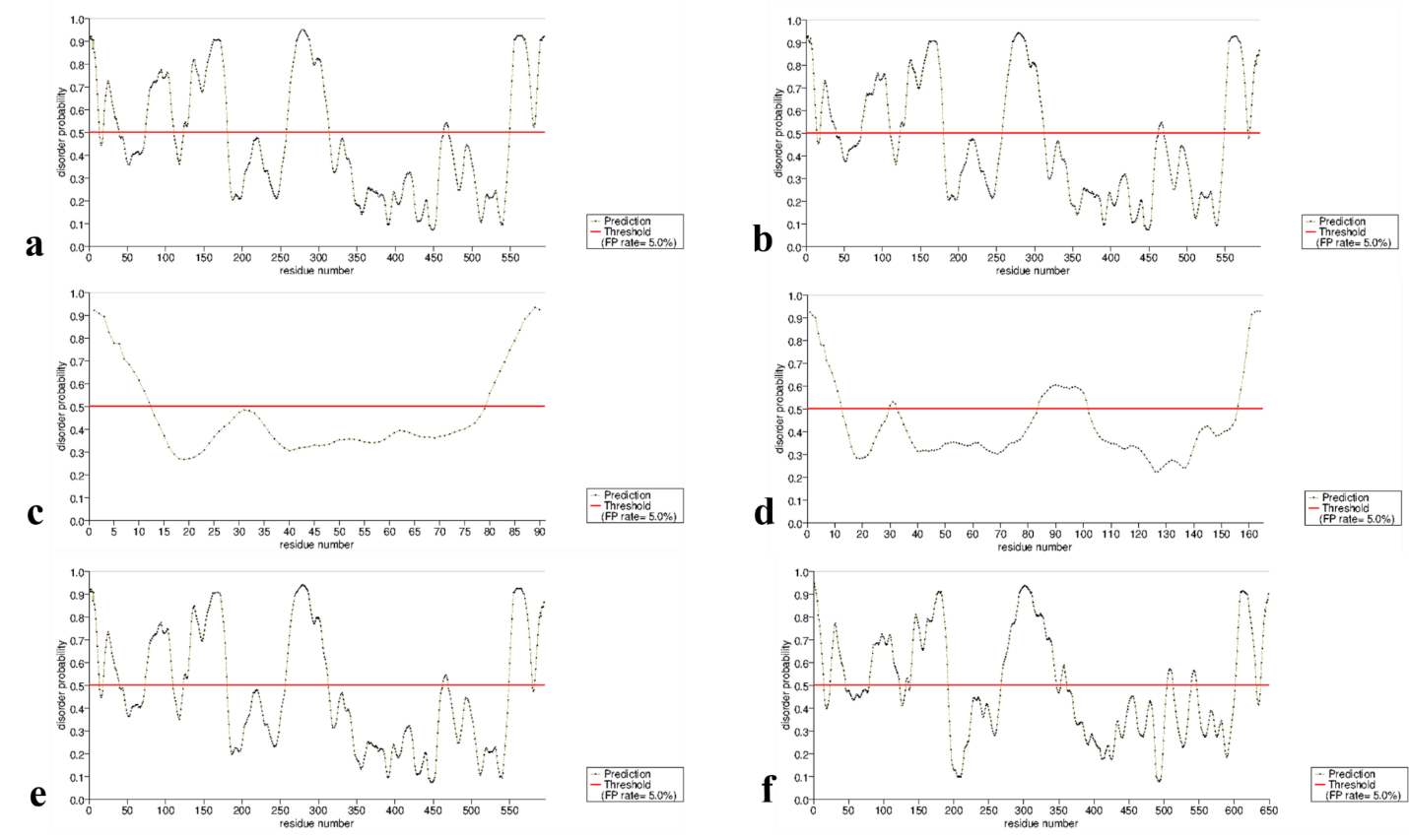
**

**
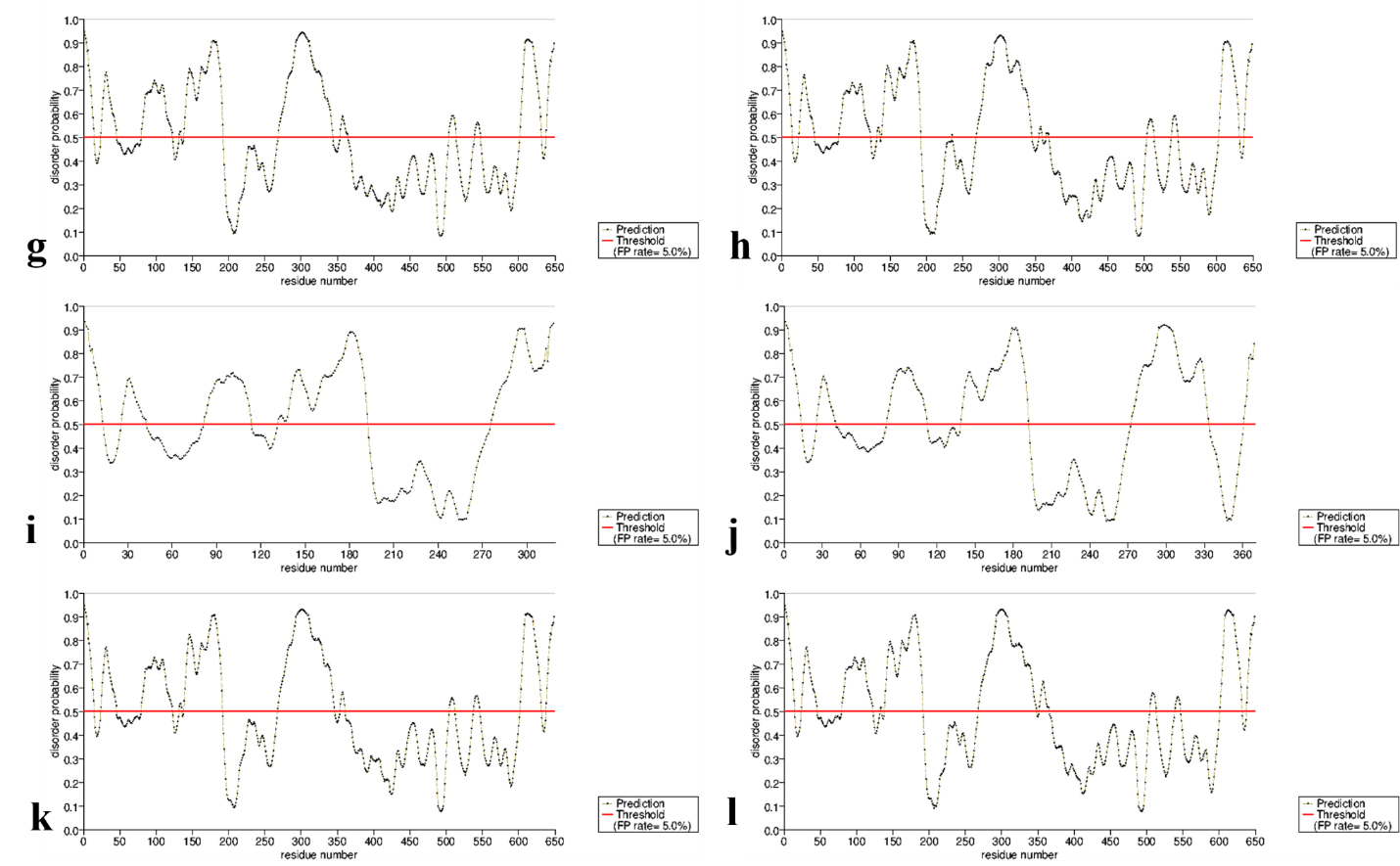
**

**
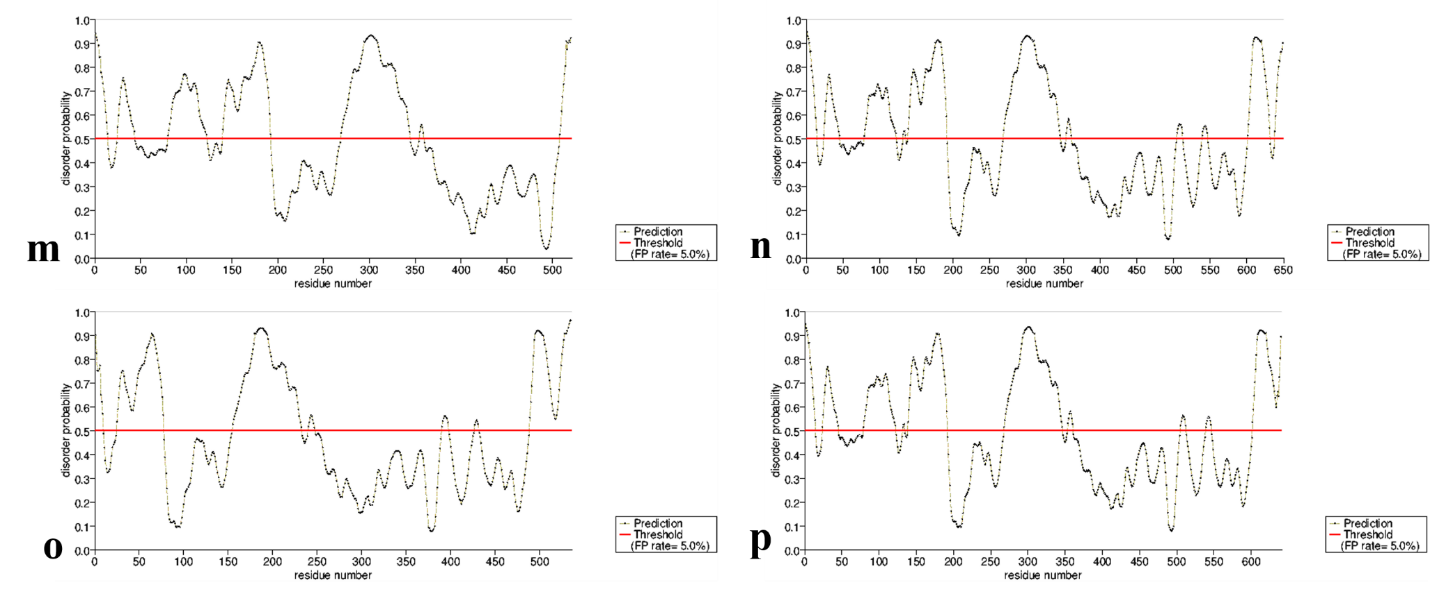
**

**Supplementary data Figure S5:** Disorder profile plots for normal and the mutated ESRα proteins analyzed in present study based on PrDOS tool

(a) normal, (b) [rs1583384537](https://asia.ensembl.org/Homo_sapiens/Variation/Summary?db=core;g=ENSG00000091831;r=6:151656691-152129619;t=ENST00000206249;vf=690836708), (c) [rs1554259481](https://asia.ensembl.org/Homo_sapiens/Variation/Summary?db=core;g=ENSG00000091831;r=6:151656691-152129619;t=ENST00000206249;vf=672757261), (d) [rs104893956](https://asia.ensembl.org/Homo_sapiens/Variation/Summary?db=core;g=ENSG00000091831;r=6:151656691-152129619;t=ENST00000206249;vf=186352285), (e) [rs761613029](https://asia.ensembl.org/Homo_sapiens/Variation/Summary?db=core;g=ENSG00000091831;r=6:151656691-152129619;t=ENST00000206249;vf=301776079), (f) [rs778449608](https://asia.ensembl.org/Homo_sapiens/Variation/Summary?db=core;g=ENSG00000091831;r=6:151656691-152129619;t=ENST00000206249;vf=314669151), (g) [rs866869178](https://asia.ensembl.org/Homo_sapiens/Variation/Summary?db=core;g=ENSG00000091831;r=6:151656691-152129619;t=ENST00000206249;vf=317238920), (h) [rs188957694](https://asia.ensembl.org/Homo_sapiens/Variation/Summary?db=core;g=ENSG00000091831;r=6:151656691-152129619;t=ENST00000206249;vf=213279813), (i) [rs755667747](https://asia.ensembl.org/Homo_sapiens/Variation/Summary?db=core;g=ENSG00000091831;r=6:151656691-152129619;t=ENST00000206249;vf=298272399), (j) [rs1467954450](https://asia.ensembl.org/Homo_sapiens/Variation/Summary?db=core;g=ENSG00000091831;r=6:151656691-152129619;t=ENST00000206249;vf=664784020), (k) [rs1584799119](https://asia.ensembl.org/Homo_sapiens/Variation/Summary?db=core;g=ENSG00000091831;r=6:151656691-152129619;t=ENST00000206249;vf=692267559), (l) [rs1131692059](https://asia.ensembl.org/Homo_sapiens/Variation/Summary?db=core;g=ENSG00000091831;r=6:151656691-152129619;t=ENST00000206249;vf=525229385), (m) [rs762742833](https://asia.ensembl.org/Homo_sapiens/Variation/Summary?db=core;g=ENSG00000091831;r=6:151656691-152129619;t=ENST00000206249;vf=302413577), (n) [rs758798083](https://asia.ensembl.org/Homo_sapiens/Variation/Summary?db=core;g=ENSG00000091831;r=6:151656691-152129619;t=ENST00000206249;vf=300171136), (o) [rs1253340312](https://asia.ensembl.org/Homo_sapiens/Variation/Summary?db=core;g=ENSG00000091831;r=6:151656691-152129619;t=ENST00000206249;vf=580549169), (p) rs143699938


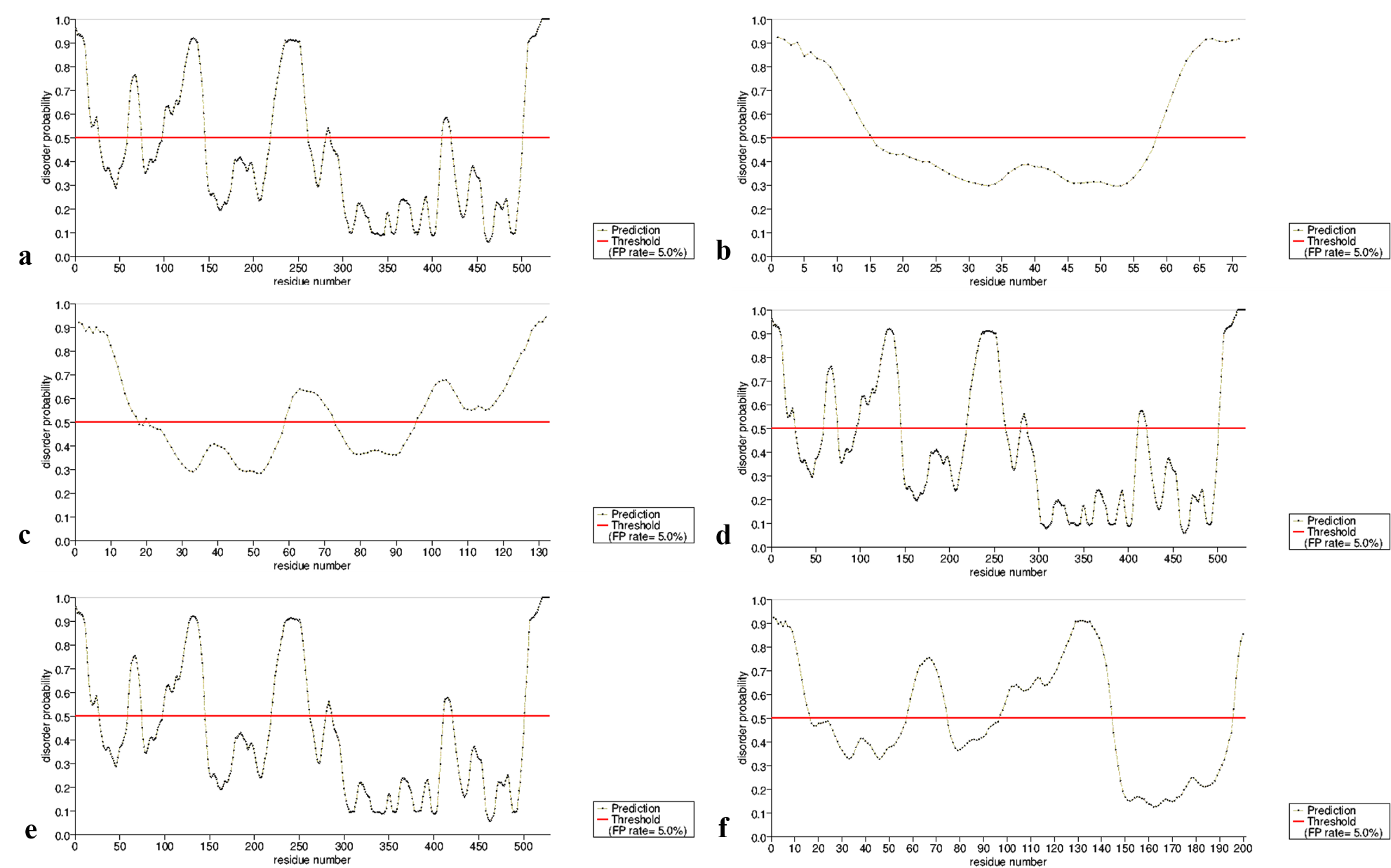


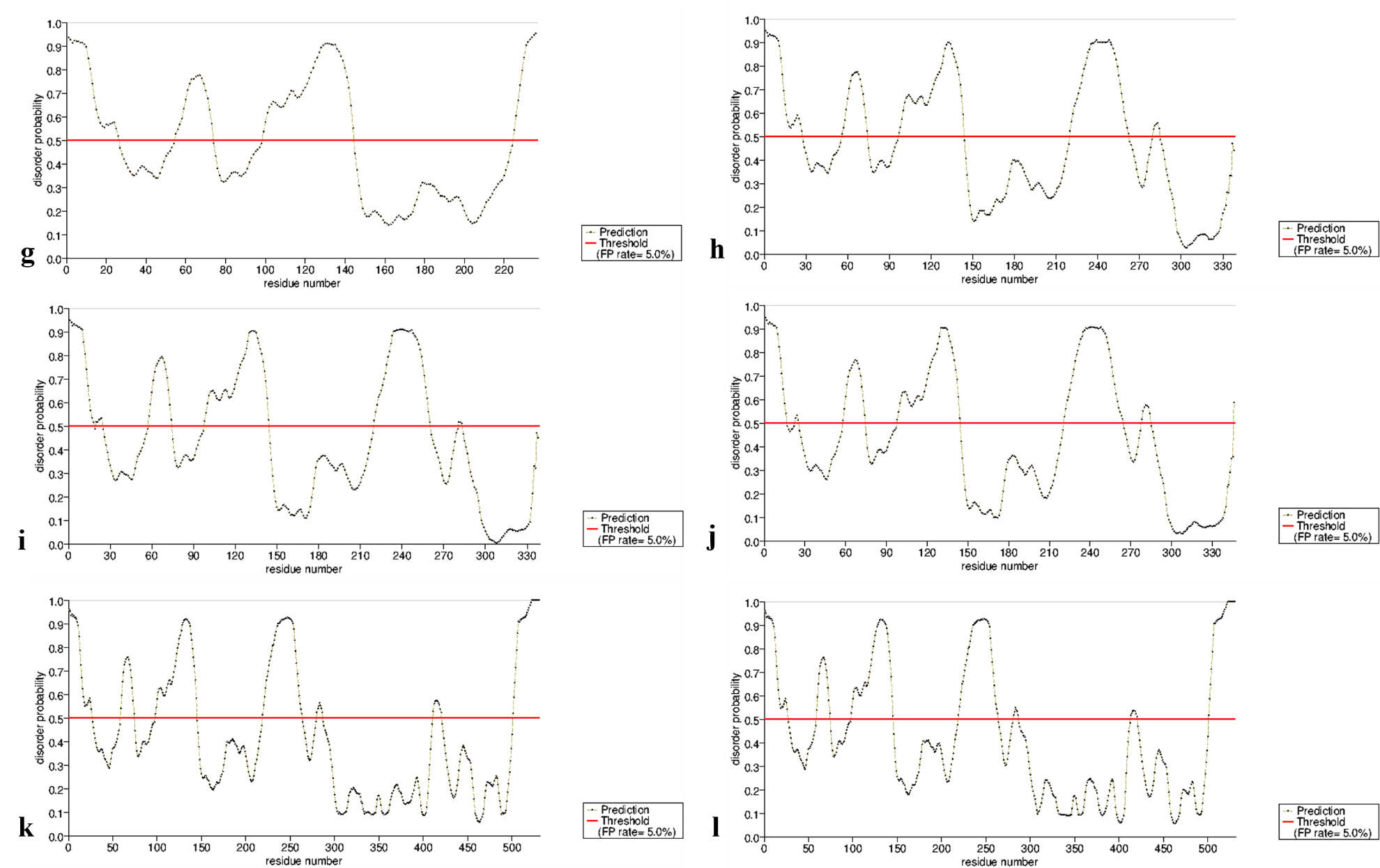


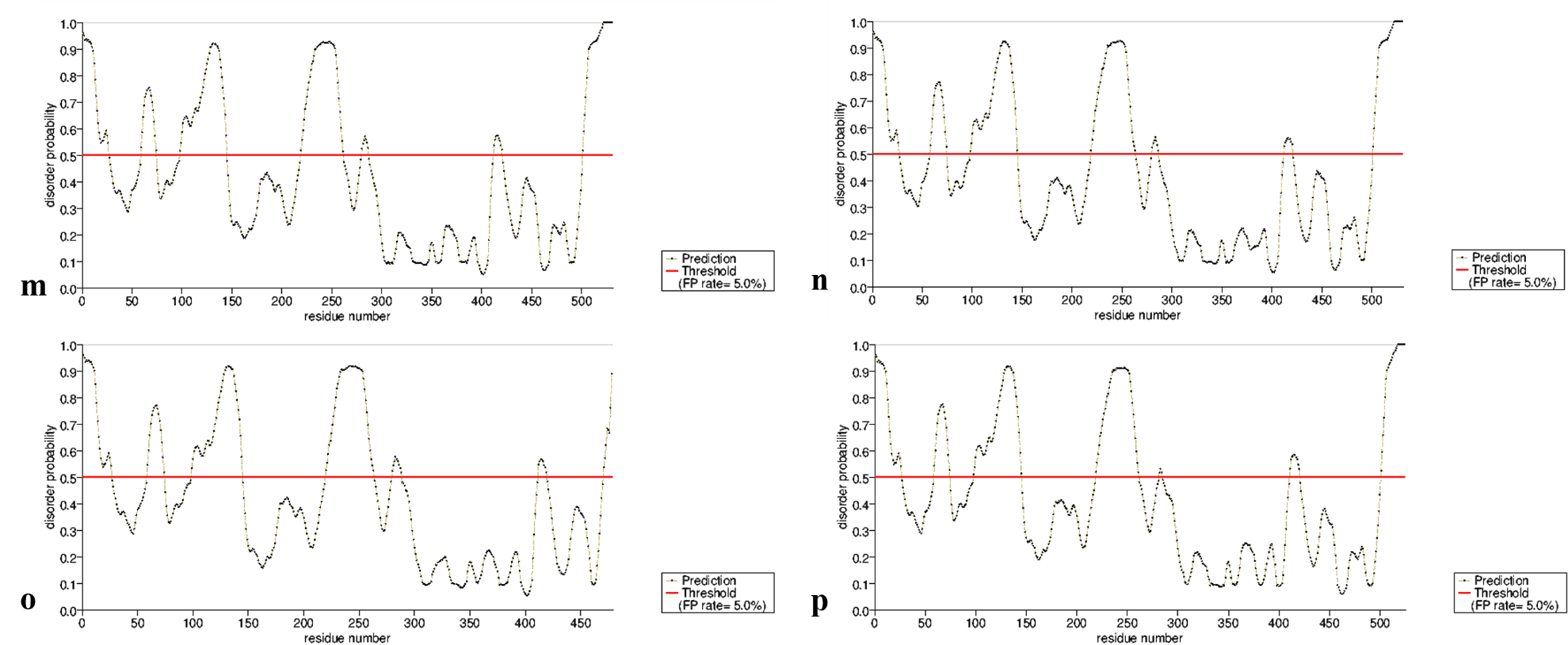


**Supplementary data Figure S6:** Disorder profile plots for normal and the mutated ESRβ proteins analyzed in present study based on PrDOS tool

(a) normal , (b) [rs1463893698](https://asia.ensembl.org/Homo_sapiens/Variation/Summary?db=core;g=ENSG00000140009;r=14:64084232-64338112;t=ENST00000341099;vf=487477622), (c) [rs140630557](https://asia.ensembl.org/Homo_sapiens/Variation/Summary?db=core;g=ENSG00000140009;r=14:64084232-64338112;t=ENST00000341099;vf=187325610), (d) [rs1450198518](https://asia.ensembl.org/Homo_sapiens/Variation/Summary?db=core;g=ENSG00000140009;r=14:64084232-64338112;t=ENST00000341099;vf=481112348), (e) [rs754945292](https://asia.ensembl.org/Homo_sapiens/Variation/Summary?db=core;g=ENSG00000140009;r=14:64084232-64338112;t=ENST00000341099;vf=252034067), (f) [rs1596423459](https://asia.ensembl.org/Homo_sapiens/Variation/Summary?db=core;g=ENSG00000140009;r=14:64084232-64338112;t=ENST00000341099;vf=523934685), (g) [rs766843910](https://asia.ensembl.org/Homo_sapiens/Variation/Summary?db=core;g=ENSG00000140009;r=14:64084232-64338112;t=ENST00000341099;vf=264787059), (h) [rs1596405923](https://asia.ensembl.org/Homo_sapiens/Variation/Summary?db=core;g=ENSG00000140009;r=14:64084232-64338112;t=ENST00000341099;vf=523929087), (i) [rs762454979](https://asia.ensembl.org/Homo_sapiens/Variation/Summary?db=core;g=ENSG00000140009;r=14:64084232-64338112;t=ENST00000341099;vf=254094829), (j) [rs1384121511](https://asia.ensembl.org/Homo_sapiens/Variation/Summary?db=core;g=ENSG00000140009;r=14:64084232-64338112;t=ENST00000341099;vf=459145327), (k) [rs1249242790](https://asia.ensembl.org/Homo_sapiens/Variation/Summary?db=core;g=ENSG00000140009;r=14:64084232-64338112;t=ENST00000341099;vf=419316481), (l) [rs1414263985](https://asia.ensembl.org/Homo_sapiens/Variation/Summary?db=core;g=ENSG00000140009;r=14:64084232-64338112;t=ENST00000341099;vf=469984932), (m) [rs78255744](https://asia.ensembl.org/Homo_sapiens/Variation/Summary?db=core;g=ENSG00000140009;r=14:64084232-64338112;t=ENST00000341099;vf=184777125), (n) [rs768924970](https://asia.ensembl.org/Homo_sapiens/Variation/Summary?db=core;g=ENSG00000140009;r=14:64084232-64338112;t=ENST00000341099;vf=265336834), (o) [rs1257844897](https://asia.ensembl.org/Homo_sapiens/Variation/Summary?db=core;g=ENSG00000140009;r=14:64084232-64338112;t=ENST00000341099;vf=428748888), (p) [rs200502775](https://asia.ensembl.org/Homo_sapiens/Variation/Summary?db=core;g=ENSG00000140009;r=14:64084232-64338112;t=ENST00000341099;vf=197621052)

**
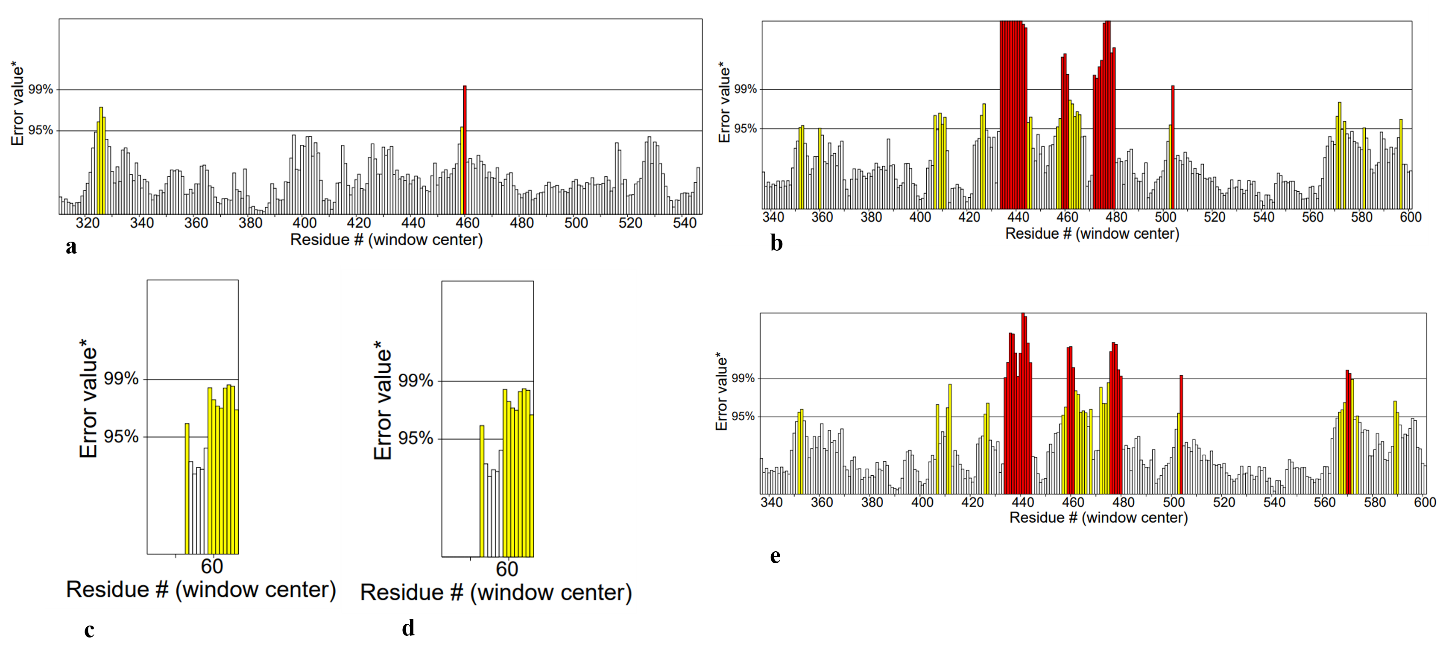
**

**
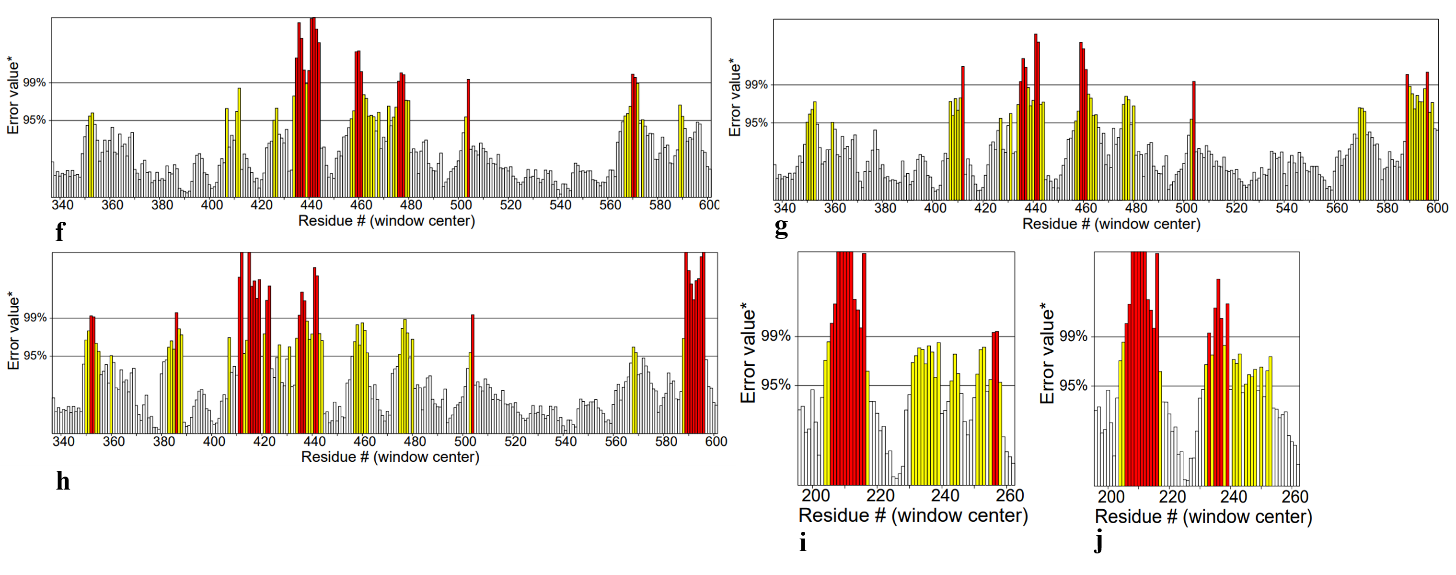
**


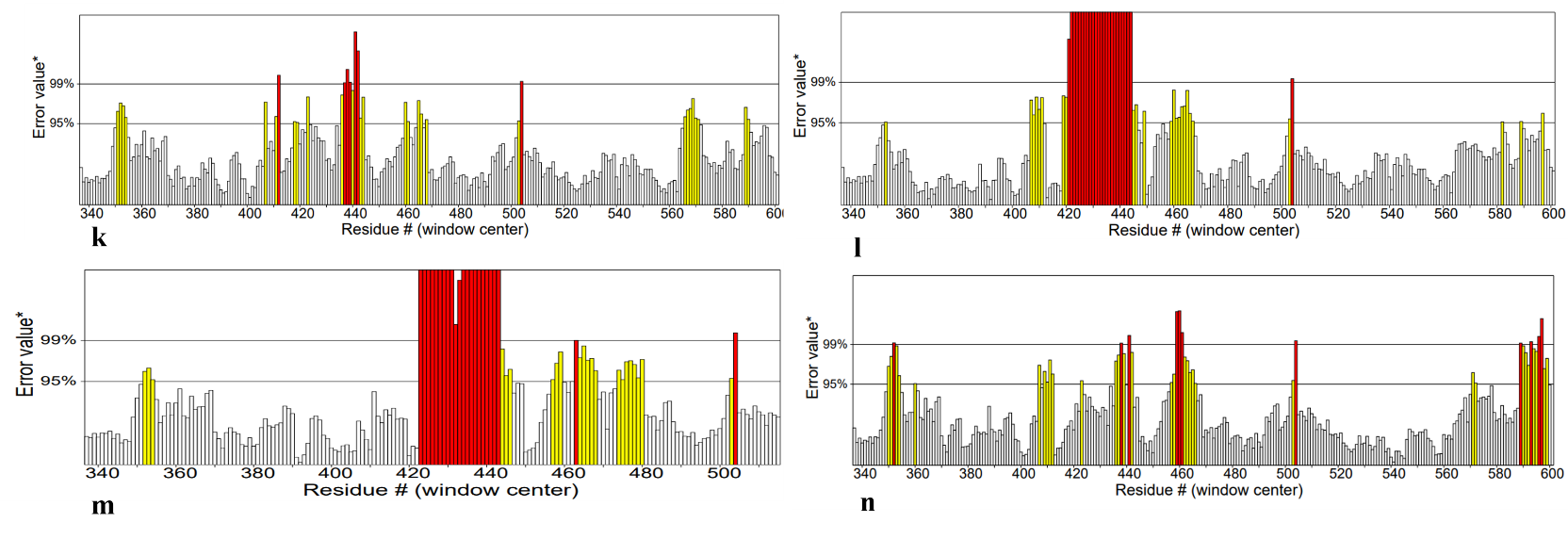


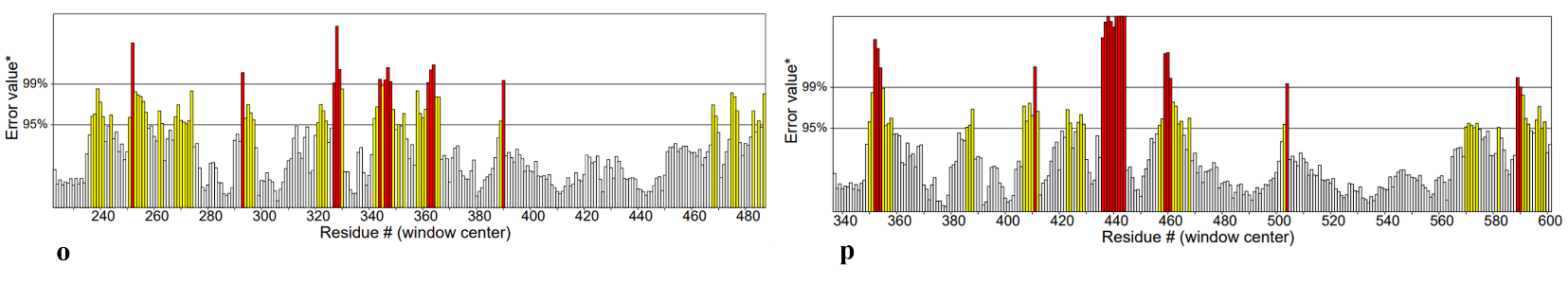


**Supplementary data Figure S7:** Validation of PHYRE2 tool generated pdb structures of normal and mutated proteins encoded by ESR1 gene using ERRAT2 tool

(a) normal, (b) [rs1583384537](https://asia.ensembl.org/Homo_sapiens/Variation/Summary?db=core;g=ENSG00000091831;r=6:151656691-152129619;t=ENST00000206249;vf=690836708), (c) [rs1554259481](https://asia.ensembl.org/Homo_sapiens/Variation/Summary?db=core;g=ENSG00000091831;r=6:151656691-152129619;t=ENST00000206249;vf=672757261), (d) [rs104893956](https://asia.ensembl.org/Homo_sapiens/Variation/Summary?db=core;g=ENSG00000091831;r=6:151656691-152129619;t=ENST00000206249;vf=186352285), (e) [rs761613029](https://asia.ensembl.org/Homo_sapiens/Variation/Summary?db=core;g=ENSG00000091831;r=6:151656691-152129619;t=ENST00000206249;vf=301776079), (f) [rs778449608](https://asia.ensembl.org/Homo_sapiens/Variation/Summary?db=core;g=ENSG00000091831;r=6:151656691-152129619;t=ENST00000206249;vf=314669151), (g) [rs866869178](https://asia.ensembl.org/Homo_sapiens/Variation/Summary?db=core;g=ENSG00000091831;r=6:151656691-152129619;t=ENST00000206249;vf=317238920), (h) [rs188957694](https://asia.ensembl.org/Homo_sapiens/Variation/Summary?db=core;g=ENSG00000091831;r=6:151656691-152129619;t=ENST00000206249;vf=213279813), (i) [rs755667747](https://asia.ensembl.org/Homo_sapiens/Variation/Summary?db=core;g=ENSG00000091831;r=6:151656691-152129619;t=ENST00000206249;vf=298272399), (j) [rs1467954450](https://asia.ensembl.org/Homo_sapiens/Variation/Summary?db=core;g=ENSG00000091831;r=6:151656691-152129619;t=ENST00000206249;vf=664784020), (k) [rs1584799119](https://asia.ensembl.org/Homo_sapiens/Variation/Summary?db=core;g=ENSG00000091831;r=6:151656691-152129619;t=ENST00000206249;vf=692267559), (l) [rs1131692059](https://asia.ensembl.org/Homo_sapiens/Variation/Summary?db=core;g=ENSG00000091831;r=6:151656691-152129619;t=ENST00000206249;vf=525229385), (m) [rs762742833](https://asia.ensembl.org/Homo_sapiens/Variation/Summary?db=core;g=ENSG00000091831;r=6:151656691-152129619;t=ENST00000206249;vf=302413577), (n) [rs758798083](https://asia.ensembl.org/Homo_sapiens/Variation/Summary?db=core;g=ENSG00000091831;r=6:151656691-152129619;t=ENST00000206249;vf=300171136), (o) [rs1253340312](https://asia.ensembl.org/Homo_sapiens/Variation/Summary?db=core;g=ENSG00000091831;r=6:151656691-152129619;t=ENST00000206249;vf=580549169), (p) [rs1436999383](https://asia.ensembl.org/Homo_sapiens/Variation/Summary?db=core;g=ENSG00000091831;r=6:151656691-152129619;t=ENST00000206249;vf=654831386)


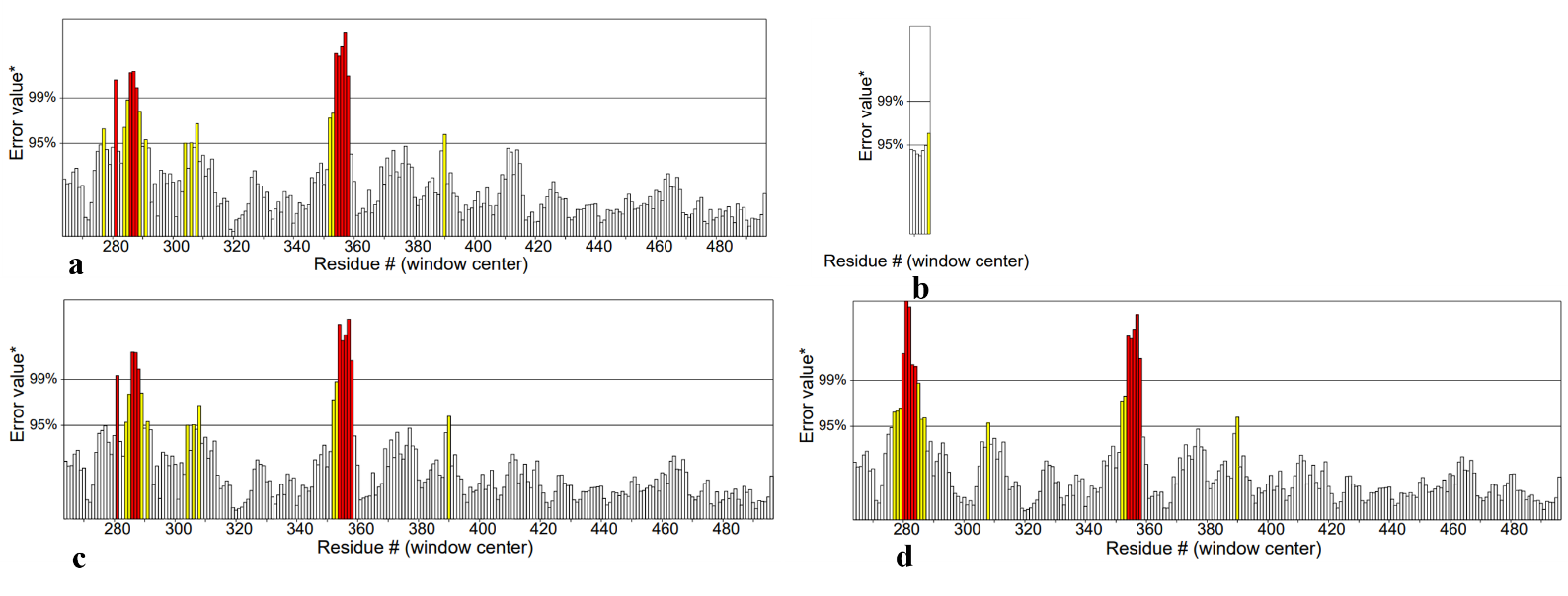


**
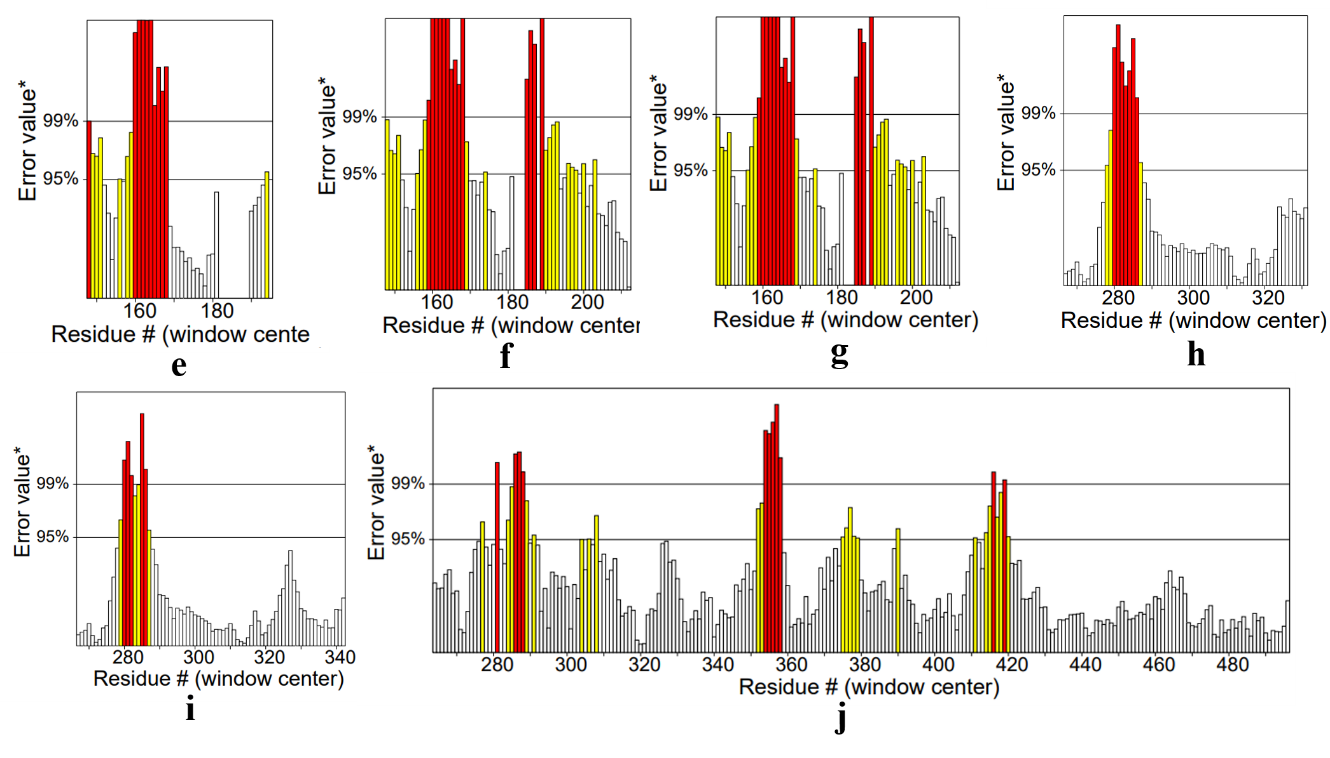
**


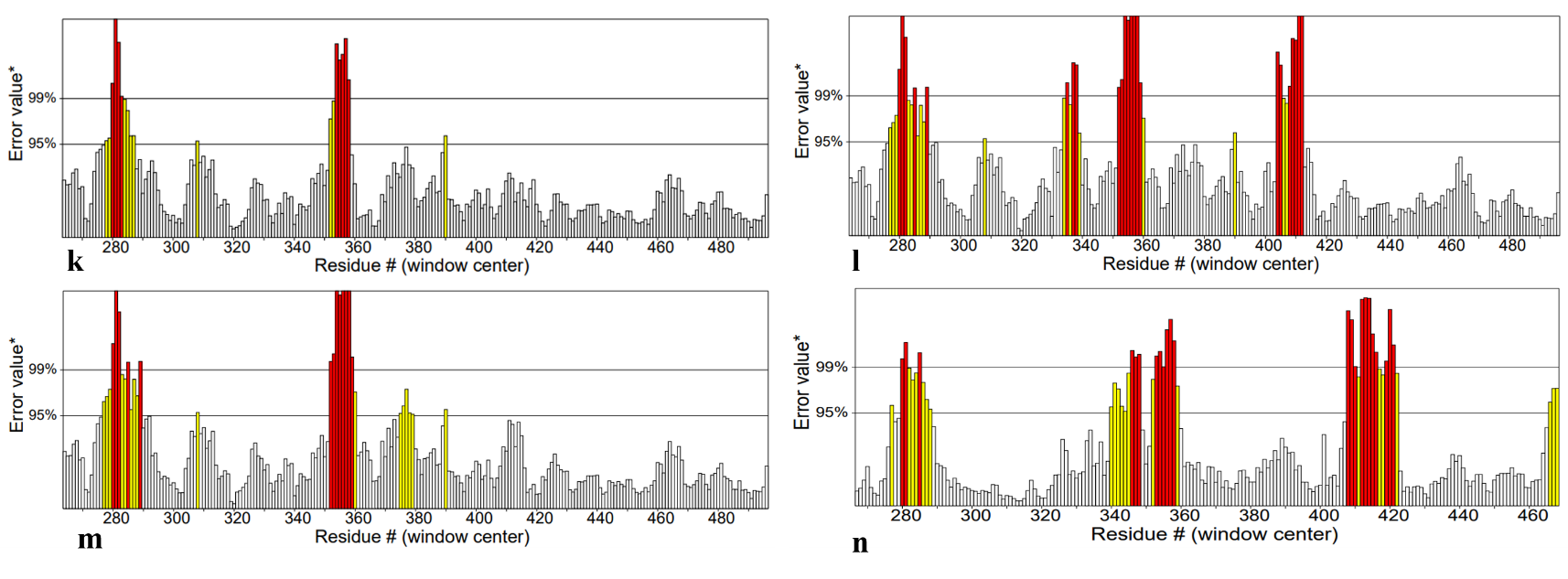


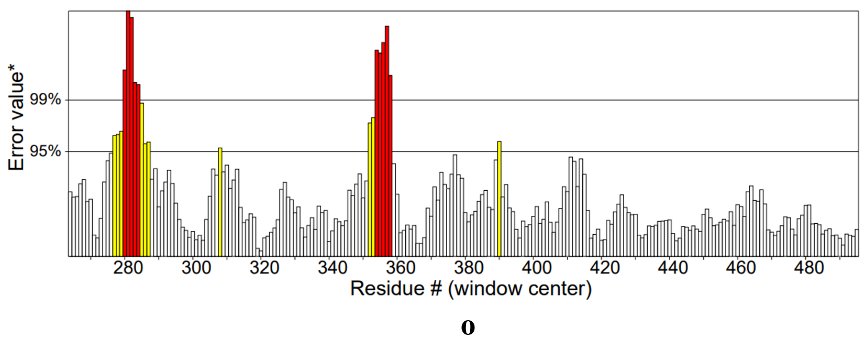


**Supplementary data Figure S8:** Validation of PHYRE2 tool generated pdb structures of normal and mutated proteins encoded by ESR2 gene using ERRAT2 tool

(a) normal , (b) [rs1463893698](https://asia.ensembl.org/Homo_sapiens/Variation/Summary?db=core;g=ENSG00000140009;r=14:64084232-64338112;t=ENST00000341099;vf=487477622), (c) [rs140630557](https://asia.ensembl.org/Homo_sapiens/Variation/Summary?db=core;g=ENSG00000140009;r=14:64084232-64338112;t=ENST00000341099;vf=187325610), (d) [rs1450198518](https://asia.ensembl.org/Homo_sapiens/Variation/Summary?db=core;g=ENSG00000140009;r=14:64084232-64338112;t=ENST00000341099;vf=481112348), (e) [rs754945292](https://asia.ensembl.org/Homo_sapiens/Variation/Summary?db=core;g=ENSG00000140009;r=14:64084232-64338112;t=ENST00000341099;vf=252034067), (f) [rs1596423459](https://asia.ensembl.org/Homo_sapiens/Variation/Summary?db=core;g=ENSG00000140009;r=14:64084232-64338112;t=ENST00000341099;vf=523934685), (g) [rs766843910](https://asia.ensembl.org/Homo_sapiens/Variation/Summary?db=core;g=ENSG00000140009;r=14:64084232-64338112;t=ENST00000341099;vf=264787059), (h) [rs1596405923](https://asia.ensembl.org/Homo_sapiens/Variation/Summary?db=core;g=ENSG00000140009;r=14:64084232-64338112;t=ENST00000341099;vf=523929087), (i) [rs762454979](https://asia.ensembl.org/Homo_sapiens/Variation/Summary?db=core;g=ENSG00000140009;r=14:64084232-64338112;t=ENST00000341099;vf=254094829), (j) [rs1384121511](https://asia.ensembl.org/Homo_sapiens/Variation/Summary?db=core;g=ENSG00000140009;r=14:64084232-64338112;t=ENST00000341099;vf=459145327), (k) [rs1249242790](https://asia.ensembl.org/Homo_sapiens/Variation/Summary?db=core;g=ENSG00000140009;r=14:64084232-64338112;t=ENST00000341099;vf=419316481), (l) [rs1414263985](https://asia.ensembl.org/Homo_sapiens/Variation/Summary?db=core;g=ENSG00000140009;r=14:64084232-64338112;t=ENST00000341099;vf=469984932), (m) [rs78255744](https://asia.ensembl.org/Homo_sapiens/Variation/Summary?db=core;g=ENSG00000140009;r=14:64084232-64338112;t=ENST00000341099;vf=184777125), (n) [rs768924970](https://asia.ensembl.org/Homo_sapiens/Variation/Summary?db=core;g=ENSG00000140009;r=14:64084232-64338112;t=ENST00000341099;vf=265336834), (o) [rs1257844897](https://asia.ensembl.org/Homo_sapiens/Variation/Summary?db=core;g=ENSG00000140009;r=14:64084232-64338112;t=ENST00000341099;vf=428748888)


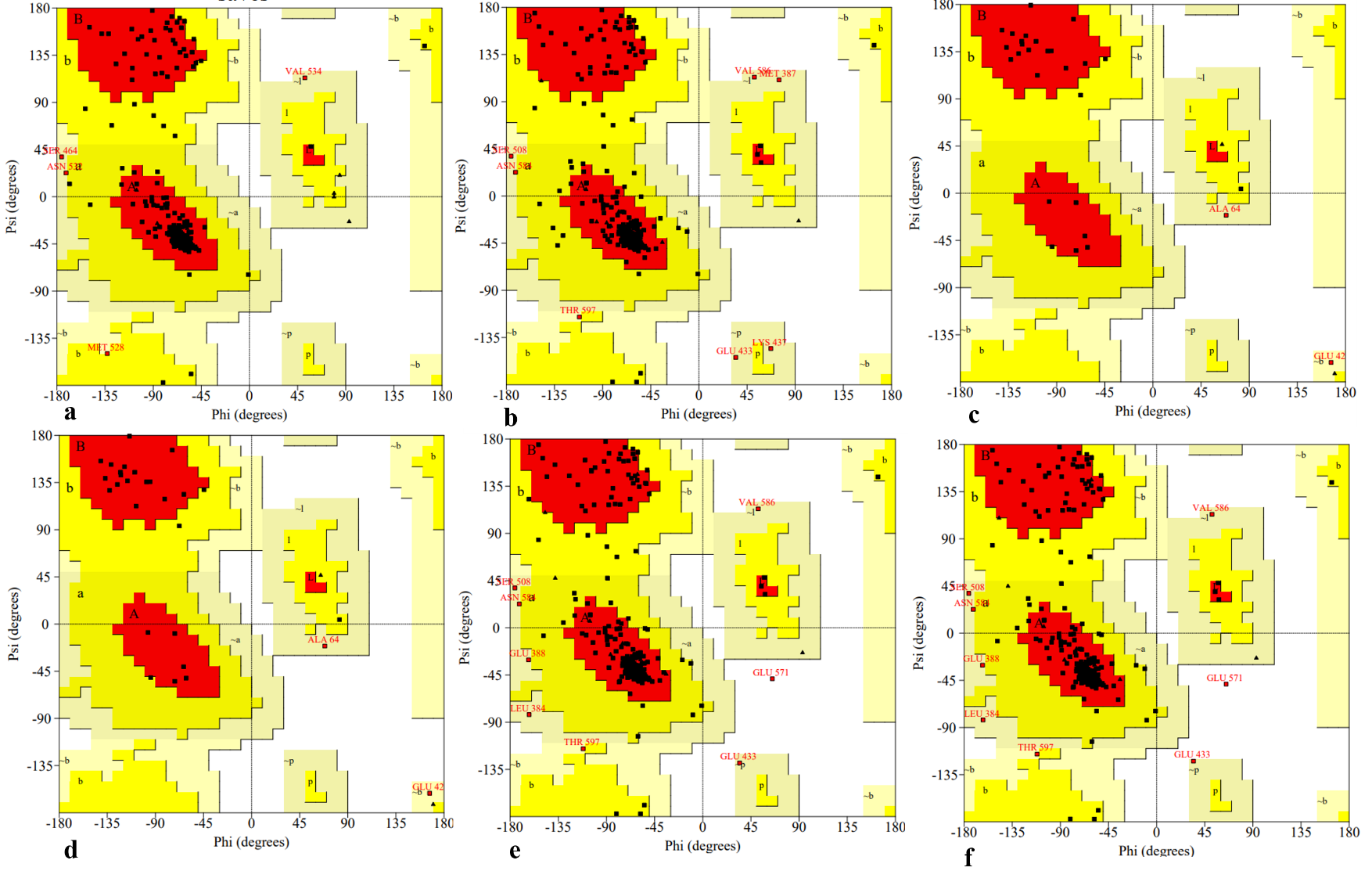


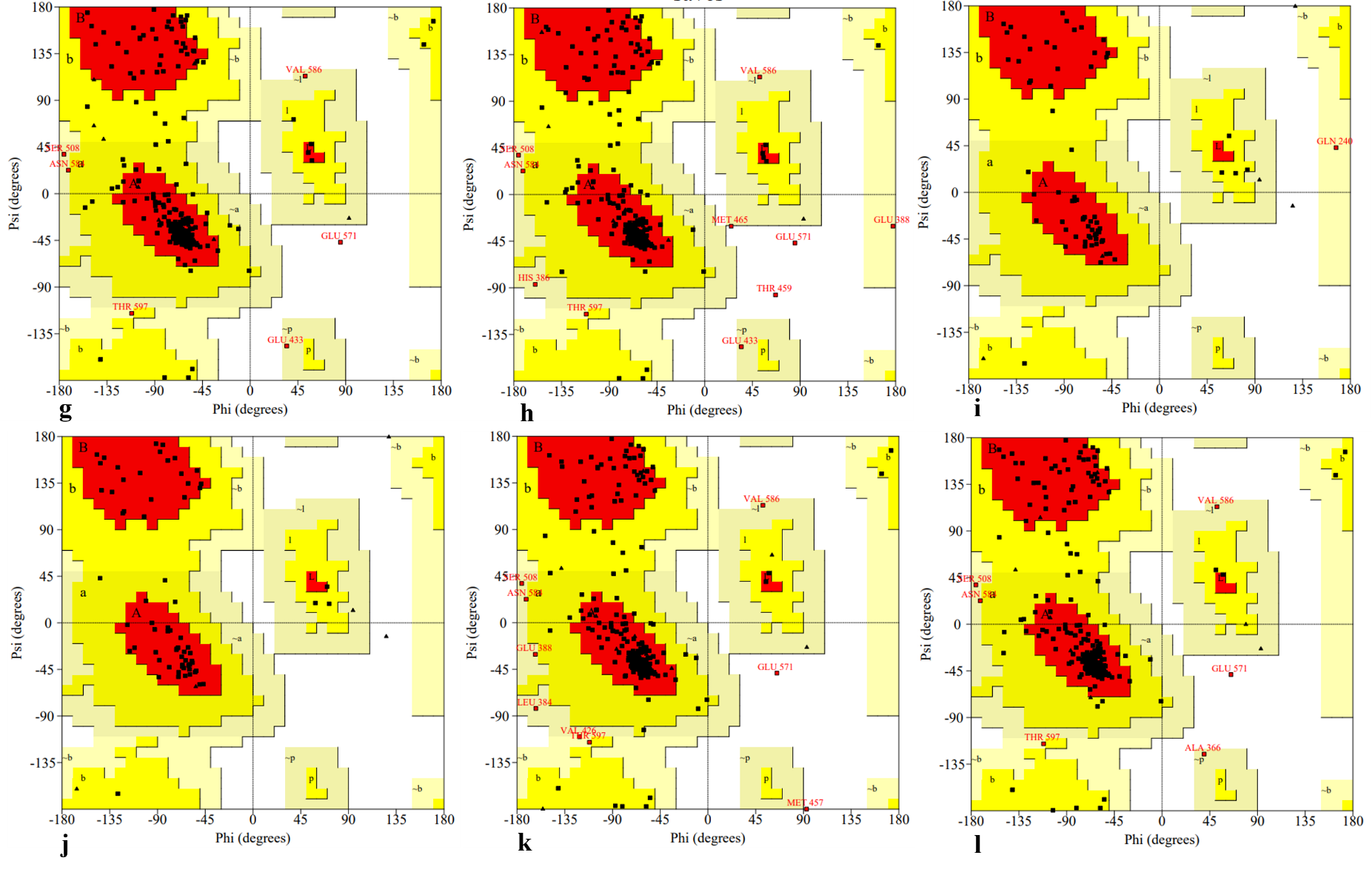


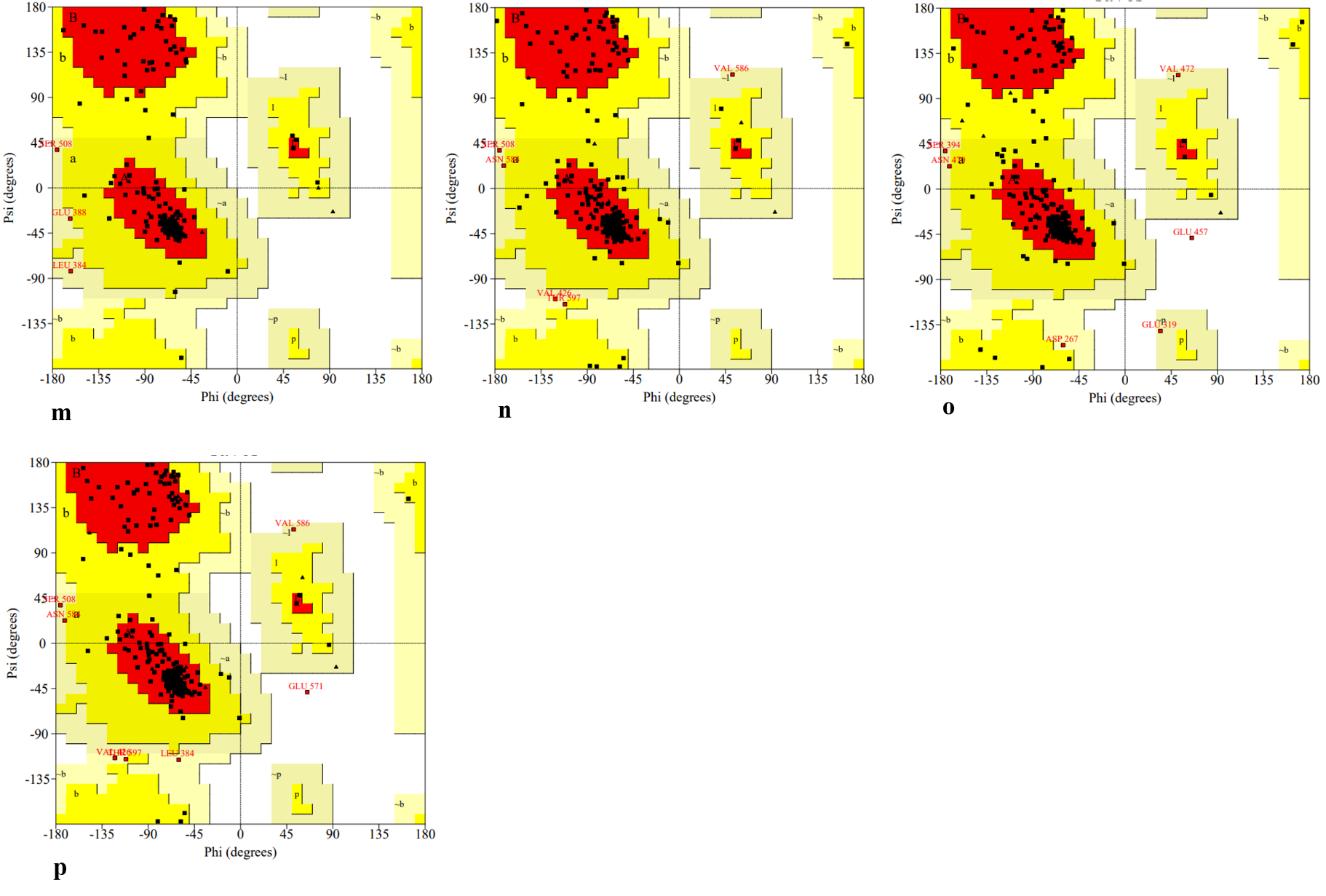


**Supplementary data Figure S9:** Validation of PHYRE2 tool generated pdb structures of normal and mutated proteins encoded by ESR1 gene using Ramachandran plots

(a) normal, (b) [rs1583384537](https://asia.ensembl.org/Homo_sapiens/Variation/Summary?db=core;g=ENSG00000091831;r=6:151656691-152129619;t=ENST00000206249;vf=690836708), (c) [rs1554259481](https://asia.ensembl.org/Homo_sapiens/Variation/Summary?db=core;g=ENSG00000091831;r=6:151656691-152129619;t=ENST00000206249;vf=672757261), (d) [rs104893956](https://asia.ensembl.org/Homo_sapiens/Variation/Summary?db=core;g=ENSG00000091831;r=6:151656691-152129619;t=ENST00000206249;vf=186352285), (e) [rs761613029](https://asia.ensembl.org/Homo_sapiens/Variation/Summary?db=core;g=ENSG00000091831;r=6:151656691-152129619;t=ENST00000206249;vf=301776079), (f) [rs778449608](https://asia.ensembl.org/Homo_sapiens/Variation/Summary?db=core;g=ENSG00000091831;r=6:151656691-152129619;t=ENST00000206249;vf=314669151), (g) [rs866869178](https://asia.ensembl.org/Homo_sapiens/Variation/Summary?db=core;g=ENSG00000091831;r=6:151656691-152129619;t=ENST00000206249;vf=317238920), (h) [rs188957694](https://asia.ensembl.org/Homo_sapiens/Variation/Summary?db=core;g=ENSG00000091831;r=6:151656691-152129619;t=ENST00000206249;vf=213279813), (i) [rs755667747](https://asia.ensembl.org/Homo_sapiens/Variation/Summary?db=core;g=ENSG00000091831;r=6:151656691-152129619;t=ENST00000206249;vf=298272399), (j) [rs1467954450](https://asia.ensembl.org/Homo_sapiens/Variation/Summary?db=core;g=ENSG00000091831;r=6:151656691-152129619;t=ENST00000206249;vf=664784020), (k) [rs1584799119](https://asia.ensembl.org/Homo_sapiens/Variation/Summary?db=core;g=ENSG00000091831;r=6:151656691-152129619;t=ENST00000206249;vf=692267559), (l) [rs1131692059](https://asia.ensembl.org/Homo_sapiens/Variation/Summary?db=core;g=ENSG00000091831;r=6:151656691-152129619;t=ENST00000206249;vf=525229385), (m) [rs762742833](https://asia.ensembl.org/Homo_sapiens/Variation/Summary?db=core;g=ENSG00000091831;r=6:151656691-152129619;t=ENST00000206249;vf=302413577), (n) [rs758798083](https://asia.ensembl.org/Homo_sapiens/Variation/Summary?db=core;g=ENSG00000091831;r=6:151656691-152129619;t=ENST00000206249;vf=300171136), (o) [rs1253340312](https://asia.ensembl.org/Homo_sapiens/Variation/Summary?db=core;g=ENSG00000091831;r=6:151656691-152129619;t=ENST00000206249;vf=580549169), (p) [rs1436999383](https://asia.ensembl.org/Homo_sapiens/Variation/Summary?db=core;g=ENSG00000091831;r=6:151656691-152129619;t=ENST00000206249;vf=654831386)

**
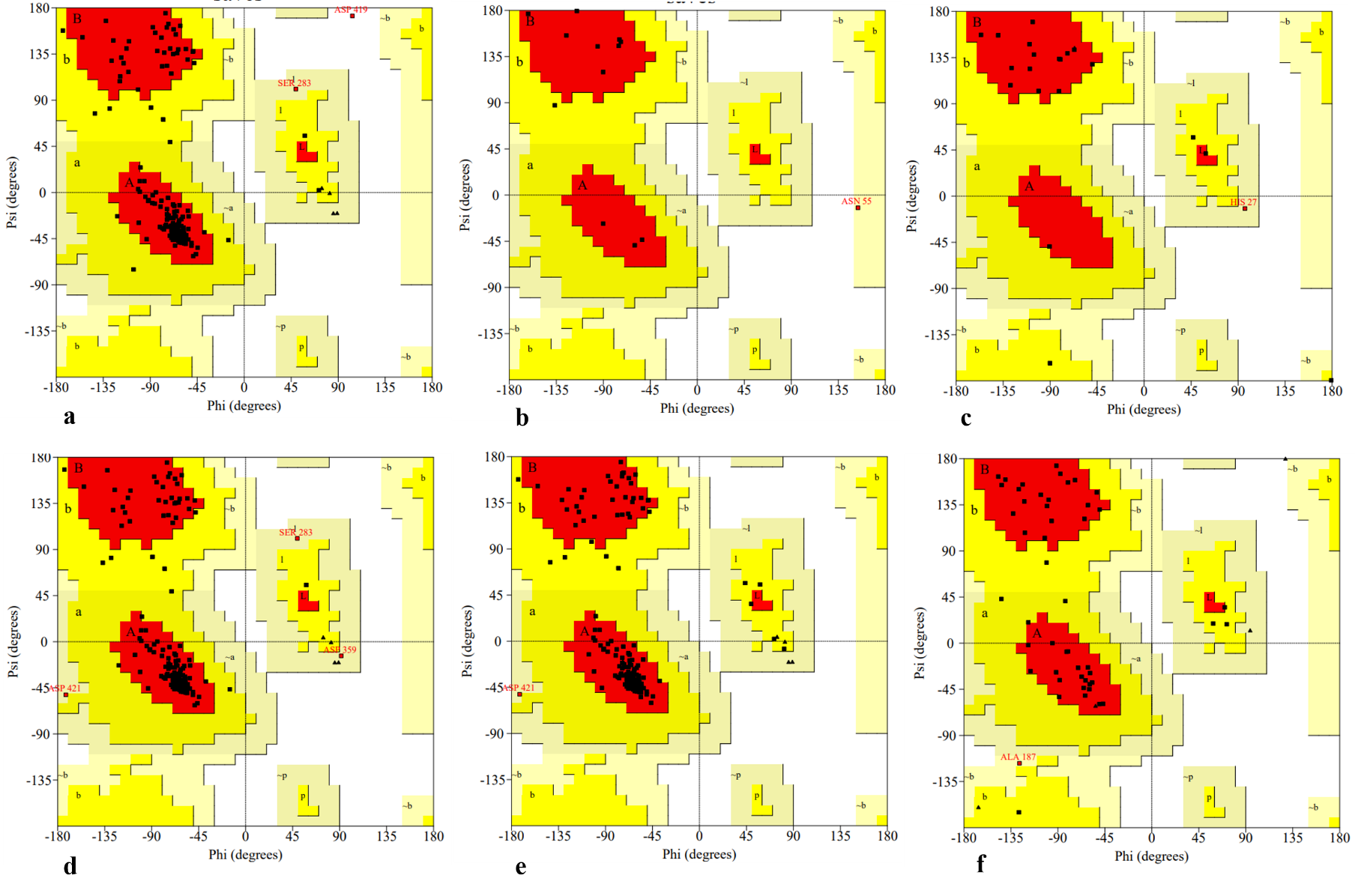
**

**
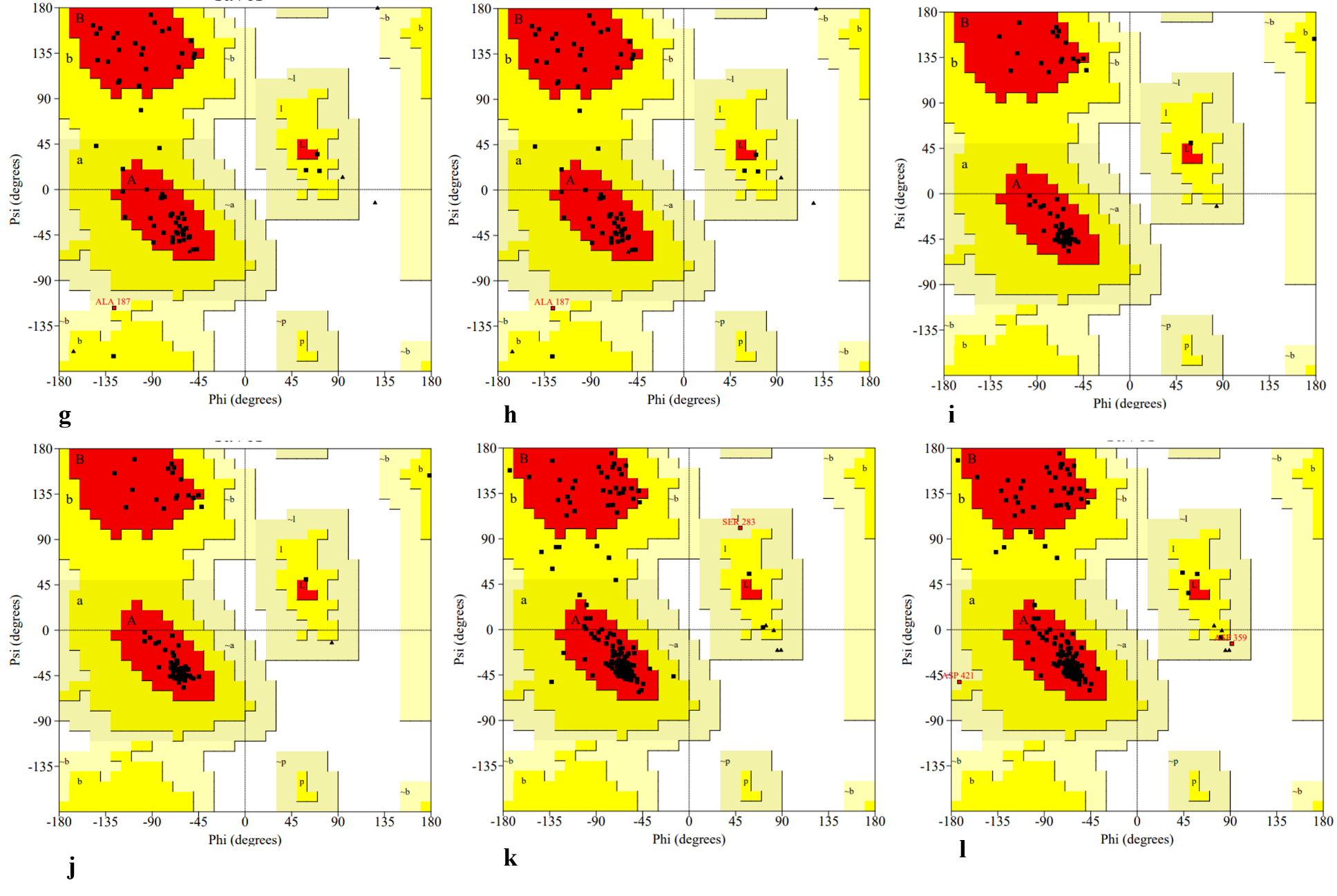
**

**
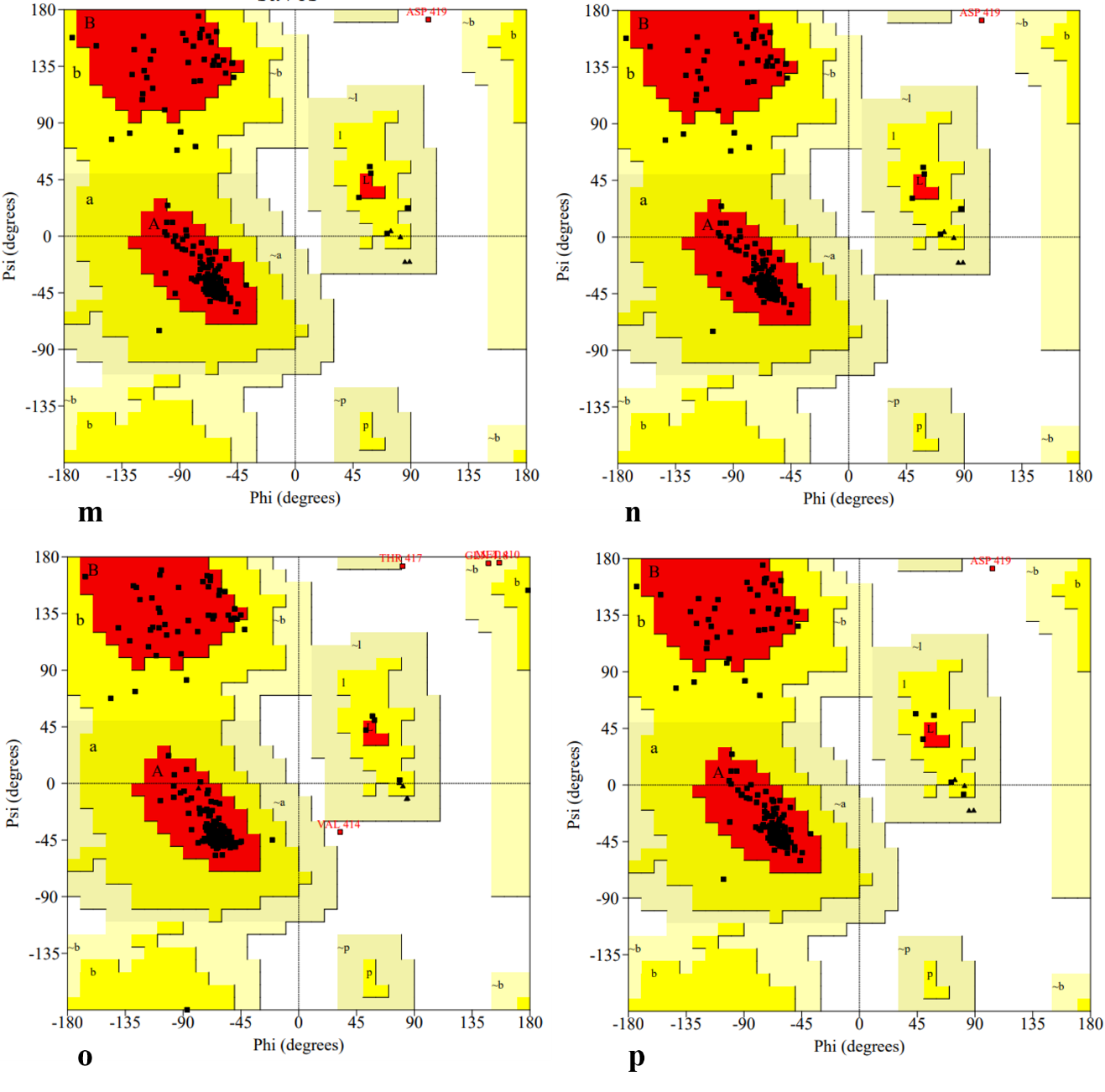
**

**Supplementary data Figure S10:** Validation of PHYRE2 tool generated pdb structures of normal and mutated proteins encoded by ESR2 gene using Ramachandran plots

(a) normal , (b) [rs1463893698](https://asia.ensembl.org/Homo_sapiens/Variation/Summary?db=core;g=ENSG00000140009;r=14:64084232-64338112;t=ENST00000341099;vf=487477622), (c) [rs140630557](https://asia.ensembl.org/Homo_sapiens/Variation/Summary?db=core;g=ENSG00000140009;r=14:64084232-64338112;t=ENST00000341099;vf=187325610), (d) [rs1450198518](https://asia.ensembl.org/Homo_sapiens/Variation/Summary?db=core;g=ENSG00000140009;r=14:64084232-64338112;t=ENST00000341099;vf=481112348), (e) [rs754945292](https://asia.ensembl.org/Homo_sapiens/Variation/Summary?db=core;g=ENSG00000140009;r=14:64084232-64338112;t=ENST00000341099;vf=252034067), (f) [rs1596423459](https://asia.ensembl.org/Homo_sapiens/Variation/Summary?db=core;g=ENSG00000140009;r=14:64084232-64338112;t=ENST00000341099;vf=523934685), (g) [rs766843910](https://asia.ensembl.org/Homo_sapiens/Variation/Summary?db=core;g=ENSG00000140009;r=14:64084232-64338112;t=ENST00000341099;vf=264787059), (h) [rs1596405923](https://asia.ensembl.org/Homo_sapiens/Variation/Summary?db=core;g=ENSG00000140009;r=14:64084232-64338112;t=ENST00000341099;vf=523929087), (i) [rs762454979](https://asia.ensembl.org/Homo_sapiens/Variation/Summary?db=core;g=ENSG00000140009;r=14:64084232-64338112;t=ENST00000341099;vf=254094829), (j) [rs1384121511](https://asia.ensembl.org/Homo_sapiens/Variation/Summary?db=core;g=ENSG00000140009;r=14:64084232-64338112;t=ENST00000341099;vf=459145327), (k) [rs1249242790](https://asia.ensembl.org/Homo_sapiens/Variation/Summary?db=core;g=ENSG00000140009;r=14:64084232-64338112;t=ENST00000341099;vf=419316481), (l) [rs1414263985](https://asia.ensembl.org/Homo_sapiens/Variation/Summary?db=core;g=ENSG00000140009;r=14:64084232-64338112;t=ENST00000341099;vf=469984932), (m) [rs78255744](https://asia.ensembl.org/Homo_sapiens/Variation/Summary?db=core;g=ENSG00000140009;r=14:64084232-64338112;t=ENST00000341099;vf=184777125), (n) [rs768924970](https://asia.ensembl.org/Homo_sapiens/Variation/Summary?db=core;g=ENSG00000140009;r=14:64084232-64338112;t=ENST00000341099;vf=265336834), (o) [rs1257844897](https://asia.ensembl.org/Homo_sapiens/Variation/Summary?db=core;g=ENSG00000140009;r=14:64084232-64338112;t=ENST00000341099;vf=428748888), (p) [rs200502775](https://asia.ensembl.org/Homo_sapiens/Variation/Summary?db=core;g=ENSG00000140009;r=14:64084232-64338112;t=ENST00000341099;vf=197621052)

**Supplementary data Table 1:** Transcript IDs and CDS of ESRα and ESRβ genes analyzed in present study

| **Gene** | **Transcript ID** | **CDS** |
| --- | --- | --- |
| ESRα | ENST00000206249.8 | ATGACCATGACCCTCCACACCAAAGCATCTGGGATGGCCCTACTGCATCAGATCCAAGGGAACGAGCTGGAGCCCCTGAACCGTCCGCAGCTCAAGATCCCCCTGGAGCGGCCCCTGGGCGAGGTGTACCTGGACAGCAGCAAGCCCGCCGTGTACAACTACCCCGAGGGCGCCGCCTACGAGTTCAACGCCGCGGCCGCCGCCAACGCGCAGGTCTACGGTCAG ACCGGCCTCCCCTACGGCCCCGGGTCTGAGGCTGCGGCGTTCGGCTCCAACGGCCTGGGGGGTTTCCCCCCACTCAACAGCGTGTCTCCGAGCCCGCTGATGCTACTGCACCCGCCGCCGCAGCTGTCGCCTTTCCTGCAGCCCCACGGCCAGCAGGTGCCCTACTACCTGGAGAACGAGCCCAGCGGCTACACGGTGCGCGAGGCCGGCCCGCCGGCATTCTACAGGCCAAATTCAGATAATCGACGCCAGGGTGGCAGAGAAAGATTGGCCAGTACCAATGACAAGGGAAGTATGGCTATGGAATCTGCCAAGGAGACTCGCTACTGTGCAGTGTGCAATGACTATGCTTCAGGCTACCATTATGGAGTCTGGTCCTGTGAGGGCTGCAAGGCCTTCTTCAAGAGAAGTATTCAAGGACATAACGACTATATGTGTCCAGCCACCAACCAG TGCACCATTGATAAAAACAGGAGGAAGAGCTGCCAGGCCTGCCGGCTCCGTAAATGCTACGAAGTGGGAATGATGAAAGGTGGGATACGAAAAGACCGAAGAGGAGGGAGATGTTGAAACACAAGCGCCAGAGAGATGATGGGGAGGGCAGGGGTGAAGTGGGGTCT GCTGGAGACATGAGAGCTGCCAACCTTTGGCCAAGCCCGCTCATGATCAAACGTCTAAGAAGAACAGCCTGGCCTTGTCCCTGACGGCCGACCAGATGGTCAGTGCCTTGTTGGATGCTGAGCCCCCGATACTCTATTCCGAGTATGATCCTACCAGACCCTTCAGTGAAGCTTCGATGATGGGCTTACTGACCAACCTGGCAGACAGGGAGCTGGTTCACATGATCAACTGGGCGAAGAGGGTGCCAGGCTTTGTGGATTTGACCCTCCATGATCAGGTCCACCTTCTAGAATGTGCCTGGCTAGAGATCCTGATGATTGGTCTCGTCTGGCGCTCCATGGAGCACCCAGGGAAGCTACTGTTTGCTCCTAACTTGCTCTTGGACAGGAACCAGGGAAAATGTGTAGAGGGCATGGTGGAGATCTTCGACATGCTGCTGGCTACATCATCTCGGTTCCGCATGATGAATCTGCAGGGAGAGGAGTTTGTGTGCCTCAAATCTATTATTTTGCTT AATTCTGGAGTGTACACATTTCTGTCCAGCACCCTGAAGTCTCTGGAAGAGAAGGACCATATCCACCGAGTCCTGGACAAGATCACAGACACTTTGATCCACCTGATGGCCAAGGCAGGCCTGACCCTGCAGCAGCAGCACCAGCGGCTGGCCCAGCTCCTCCTCATCCTCTCCCACATCAGGCACATGAGTAACAAAGGCATGGAGCATCTGTACAGCATGAAGTGCAAGAACGTGGTGCCCCTCTATGACCTGCTGCTGGAGATGCTGGACGCCCACCGCCTACATGCGCCCACTAGCCGTGGAGGGGCATCCGTGGAGGAGACGGACCAAAGCCACTTGGCCACTGCGGGCTCTACTTCACGCATTCCTTGCAAAAGTATTACATCACGGGGGAGGCAGAGGGTTTCCCT GCCACGGTCTGA |
| ESRβ | ENST00000341099.6 | ATGGATATAAAAAACTCACCATCTAGCCTTAATTCTCCTTCCTCCTACAACTGCAGTCAATCCATCTTACCCCTGGAGCACGGCTCCATATACATACCTTCCTCCTATGTAGACAGCCACCATGAATATCCAGCCATGACATTCTATAGCCCTGCTGTGATGAATTACAGCATTCCCAGCAATGTCACTAACTTGGAAGGTGGGCCTGGTCGGCAGACCACAAGCCCAAATGTGTTGTGGCCAACACCTGGGCACCTTTCTCCTTTAGTGGTCCATCGCCAGTTATCACATCTGTATGCGGAACCTCAAAAGAGTCCCTGGTGTGAAGCAAGATCGCTAGAACACACCTTACCTGTAAACAGAGAGACACTGAAAAGGAAGGTTAGTGGGAACCGTTGCGCCAGCCCTGTTACTGGTCCAGGTTCAAAGAGGGATGCTCACTTCTGCGCTGTCTGCAGCGATTACGCATCGGGATATCACTATGGAGTCTGGTCGTGTGAAGGATGTAAGGCCTTTTTTAAAAGAAGCATTCAAGGACATAATGATTATATTTGTCCAGCTACAAATCAGTGTACAATCGATAAAAACCGGCGCAAGAGCTGCCAGGCCTGCCGACTTCGGAAGTGTTACGAAGTGGGAATGGTGAAGTGTGGCTCCCGGAGAGAGAGATGTGGGTACCGCCTTGTGCGGAGACAGAGAAGTGCCGACGAGCAGCTGCACTGTGCCGGCAAGGCCAAGAGAAGTGGCGGCCACGCGCCCCGAGTGCGGGAGCTGCTGCTGGACGCCCTGAGCCCCGAGCAGCTAGTGCTCACCCTCCTGGAGGCTGAGCCGCCCCATGTGCTGATCAGCCGCCCCAGTGCGCCCTTCACCGAGGCCTCCATGATGATGTCCCTGACCAAGTTGGCCGACAAGGAGTTGGTACACATGATCAGCTGGGCCAAGAAGATTCCCGGCTTTGTGGAGCTCAGCCTGTTCGACCAAGTGCGGCTCTTGGAGAGCTGTTGGATGGAGGTGTTAATGATGGGGCTGATGTGGCGCTCAATTGACCACCCCGGCAAGCTCATCTTTGCTCCAGATCTTGTTCTGGACAGGGATGAGGGGAAATGCGTAGAAGGAATTCTGGAAATCTTTGACATGCTCCTGGCAACTACTTCAAGGTTTCGAGAGTTAAAACTCCAACACAAAGAATATCTCTGTGTCAAGGCCATGATCCTGCTCAATTCCAGTATGTACCCTCTGGTCACAGCGACCCAGGATGCTGACAGCAGCCGGAAGCTGGCTCACTTGCTGAACGCCGTGACCGATGCTTTGGTTTGGGTGATTGCCAAGAGCGGCATCTCCTCCCAGCAGCAATCCATGCGCCTGGCTAACCTCCTGATGCTCCTGTCCCACGTCAGGCATGCGAGTAACAAGGGCATGGAACATCTGCTCAACATGAAGTGCAAAAATGTGGTCCCAGTGTATGACCTGCTGCTGGAGATGCTGAATGCCCACGTGCTTCGCGGGTGCAAGTCCTCCATCACGGGGTCCGAGTGCAGCCCGGCAGAGGACAGTAAAAGCAAAGAGGGCTCCCAGAACCCACAGTCTCAGTGA |

**Supplementary data Table 2:** Predicting the effect of SNPs on number of disordered regions and the number of disordered amino acids of ESRα and ESRβ genes predicted using PrDOS server

| **#** | **SNPs ID** | **No. of disordered regions** | **No. of disordered amino acids** |
| --- | --- | --- | --- |
| ESR1 | | | |
| 1 | Normal | 137 | 7 |
| 2 | [rs1583384537](https://asia.ensembl.org/Homo_sapiens/Variation/Summary?db=core;g=ENSG00000091831;r=6:151656691-152129619;t=ENST00000206249;vf=690836708) | 130 | 7 |
| 3 | [rs1554259481](https://asia.ensembl.org/Homo_sapiens/Variation/Summary?db=core;g=ENSG00000091831;r=6:151656691-152129619;t=ENST00000206249;vf=672757261) | 23 | 2 |
| 4 | [rs104893956](https://asia.ensembl.org/Homo_sapiens/Variation/Summary?db=core;g=ENSG00000091831;r=6:151656691-152129619;t=ENST00000206249;vf=186352285) | 42 | 4 |
| 5 | [rs761613029](https://asia.ensembl.org/Homo_sapiens/Variation/Summary?db=core;g=ENSG00000091831;r=6:151656691-152129619;t=ENST00000206249;vf=301776079) | 234 | 7 |
| 6 | [rs778449608](https://asia.ensembl.org/Homo_sapiens/Variation/Summary?db=core;g=ENSG00000091831;r=6:151656691-152129619;t=ENST00000206249;vf=314669151) | 283 | 11 |
| 7 | [rs866869178](https://asia.ensembl.org/Homo_sapiens/Variation/Summary?db=core;g=ENSG00000091831;r=6:151656691-152129619;t=ENST00000206249;vf=317238920) | 284 | 11 |
| 8 | [rs188957694](https://asia.ensembl.org/Homo_sapiens/Variation/Summary?db=core;g=ENSG00000091831;r=6:151656691-152129619;t=ENST00000206249;vf=213279813) | 286 | 13 |
| 9 | [rs755667747](https://asia.ensembl.org/Homo_sapiens/Variation/Summary?db=core;g=ENSG00000091831;r=6:151656691-152129619;t=ENST00000206249;vf=298272399) | 166 | 5 |
| 10 | [rs1467954450](https://asia.ensembl.org/Homo_sapiens/Variation/Summary?db=core;g=ENSG00000091831;r=6:151656691-152129619;t=ENST00000206249;vf=664784020) | 184 | 6 |
| 11 | [rs1584799119](https://asia.ensembl.org/Homo_sapiens/Variation/Summary?db=core;g=ENSG00000091831;r=6:151656691-152129619;t=ENST00000206249;vf=692267559) | 180 | 11 |
| 12 | [rs1131692059](https://asia.ensembl.org/Homo_sapiens/Variation/Summary?db=core;g=ENSG00000091831;r=6:151656691-152129619;t=ENST00000206249;vf=525229385) | 289 | 11 |
| 13 | [rs762742833](https://asia.ensembl.org/Homo_sapiens/Variation/Summary?db=core;g=ENSG00000091831;r=6:151656691-152129619;t=ENST00000206249;vf=302413577) | 223 | 7 |
| 14 | [rs758798083](https://asia.ensembl.org/Homo_sapiens/Variation/Summary?db=core;g=ENSG00000091831;r=6:151656691-152129619;t=ENST00000206249;vf=300171136) | 282 | 11 |
| 15 | [rs1253340312](https://asia.ensembl.org/Homo_sapiens/Variation/Summary?db=core;g=ENSG00000091831;r=6:151656691-152129619;t=ENST00000206249;vf=580549169) | 208 | 7 |
| 16 | [rs1436999383](https://asia.ensembl.org/Homo_sapiens/Variation/Summary?db=core;g=ENSG00000091831;r=6:151656691-152129619;t=ENST00000206249;vf=654831386) | 277 | 10 |
| ESR2 | | | |
| 1 | Normal | 177 | 7 |
| 2 | [rs1463893698](https://asia.ensembl.org/Homo_sapiens/Variation/Summary?db=core;g=ENSG00000140009;r=14:64084232-64338112;t=ENST00000341099;vf=487477622) | 28 | 2 |
| 3 | [rs140630557](https://asia.ensembl.org/Homo_sapiens/Variation/Summary?db=core;g=ENSG00000140009;r=14:64084232-64338112;t=ENST00000341099;vf=187325610) | 69 | 3 |
| 4 | [rs1450198518](https://asia.ensembl.org/Homo_sapiens/Variation/Summary?db=core;g=ENSG00000140009;r=14:64084232-64338112;t=ENST00000341099;vf=481112348) | 183 | 7 |
| 5 | [rs754945292](https://asia.ensembl.org/Homo_sapiens/Variation/Summary?db=core;g=ENSG00000140009;r=14:64084232-64338112;t=ENST00000341099;vf=252034067) | 177 | 7 |
| 6 | [rs1596423459](https://asia.ensembl.org/Homo_sapiens/Variation/Summary?db=core;g=ENSG00000140009;r=14:64084232-64338112;t=ENST00000341099;vf=523934685) | 86 | 4 |
| 7 | [rs766843910](https://asia.ensembl.org/Homo_sapiens/Variation/Summary?db=core;g=ENSG00000140009;r=14:64084232-64338112;t=ENST00000341099;vf=264787059) | 103 | 4 |
| 8 | [rs1596405923](https://asia.ensembl.org/Homo_sapiens/Variation/Summary?db=core;g=ENSG00000140009;r=14:64084232-64338112;t=ENST00000341099;vf=523929087) | 140 | 5 |
| 9 | [rs762454979](https://asia.ensembl.org/Homo_sapiens/Variation/Summary?db=core;g=ENSG00000140009;r=14:64084232-64338112;t=ENST00000341099;vf=254094829) | 131 | 6 |
| 10 | [rs1384121511](https://asia.ensembl.org/Homo_sapiens/Variation/Summary?db=core;g=ENSG00000140009;r=14:64084232-64338112;t=ENST00000341099;vf=459145327) | 135 | 7 |
| 11 | [rs1249242790](https://asia.ensembl.org/Homo_sapiens/Variation/Summary?db=core;g=ENSG00000140009;r=14:64084232-64338112;t=ENST00000341099;vf=419316481) | 181 | 7 |
| 12 | [rs1414263985](https://asia.ensembl.org/Homo_sapiens/Variation/Summary?db=core;g=ENSG00000140009;r=14:64084232-64338112;t=ENST00000341099;vf=469984932) | 189 | 7 |
| 13 | [rs78255744](https://asia.ensembl.org/Homo_sapiens/Variation/Summary?db=core;g=ENSG00000140009;r=14:64084232-64338112;t=ENST00000341099;vf=184777125) | 178 | 7 |
| 14 | [rs768924970](https://asia.ensembl.org/Homo_sapiens/Variation/Summary?db=core;g=ENSG00000140009;r=14:64084232-64338112;t=ENST00000341099;vf=265336834) | 182 | 7 |
| 15 | [rs1257844897](https://asia.ensembl.org/Homo_sapiens/Variation/Summary?db=core;g=ENSG00000140009;r=14:64084232-64338112;t=ENST00000341099;vf=428748888) | 157 | 7 |
| 16 | [rs200502775](https://asia.ensembl.org/Homo_sapiens/Variation/Summary?db=core;g=ENSG00000140009;r=14:64084232-64338112;t=ENST00000341099;vf=197621052) | 170 | 7 |
